# Binding and Flip as Initial Steps for BP-100 Antimicrobial Actions

**DOI:** 10.1038/s41598-019-45075-5

**Published:** 2019-06-13

**Authors:** Peter Park, Leandro R. Franco, Hernan Chaimovich, Kaline Coutinho, Iolanda M. Cuccovia, Filipe S. Lima

**Affiliations:** 1 Departamento de Bioquímica, Instituto de Química, Universidade de São Paulo, São Paulo, Brazil; 2 Departamento de Física Geral, Instituto de Física, Universidade de São Paulo, São Paulo, Brazil; 3 Departamento de Química Fundamental, Centro de Ciências Exatas e da Natureza, Universidade Federal de Pernambuco, Recife, Brazil

**Keywords:** Membrane biophysics, Computational biophysics

## Abstract

BP100 is a short antimicrobial peptide and can also act as a molecule-carrier into cells. Like with other antimicrobial peptides, the precise mechanism of membrane disruption is not fully understood. Here we use computer simulations to understand, at a molecular level, the initial interaction between BP100 and zwitterionic/negatively charged model membranes. In agreement with experimental results, our simulations showed BP100 folded into an alpha helix when in contact with negatively charged membranes. BP100 binding induced the aggregation of negatively charged lipids on mixed membranes composed of zwitterionic and anionic lipids. The peptide in alpha-helix conformation initially interacts with the membrane via electrostatic interactions between the negatively charged lipids and the positively charged residues of the peptide. At that point the peptide flips, burying the hydrophobic residues into the bilayer highlighting the importance of the hydrophobic effect contribution to the initial interaction of cationic antimicrobial peptides with membranes.

## Introduction

Cationic antimicrobial peptides (CAMPs) are short-length, amphiphilic, rich in basic residues of the innate immune system of complex organisms, serving as the first defense line against pathogens. CAMPs can potentially give rise to a new generation of drugs due to their broad spectrum of antimicrobial activity against bacteria, rare cases of the appearance of AMP-resistant bacteria, selectivity, and rapid effects. Due to their potential advantage as antimicrobials, new AMPs are increasingly being isolated or synthesized and their properties investigated. Although increasing evidence shows that some AMPs may also act intracellularly, inhibiting proteins^1^, nucleic acids^1–4^ and cell-wall synthesis^1–5^ and inducing cell apoptosis^1,3,5^, most of them kill cells by disturbing the membrane’s integrity^1,2,6,7^. Several CAMPs seem to disturb the membrane of cells by self-aggregation on the membrane and forming pores beyond a peptide/lipid (P/L) ratio threshold^1,2^.

BP100 (H-KKLFKKILKYL-NH_2_), is a hybrid CAMP synthesized by combinatorial chemistry involving parts of two natural AMPs, melittin, and cecropin A^8^. BP100 shows high antimicrobial activity and selectivity against some Gram-negative bacteria, with a promising minimal inhibitory concentration ranging from 2.5 to 5 µM and low cytotoxicity against mammalian cells making this peptide an exciting candidate for therapeutic use^8^. BP100 conformation and activity on model membranes have been extensively analyzed with experimental techniques^9–13^. BP100 has a random coil conformation in solution and zwitterionic bilayers but acquires predominantly alpha-helical and amphipathic conformation in the presence of negatively charged membranes. BP100 is highly mobile on the membrane surface and orients parallel in predominantly negatively charged vesicles^9,10^. BP100 secondary structure formation and bacteria killing performance seem to be directly related to negative charge content in membranes, thus explaining its high selectivity towards negative bacterial membrane and low cytotoxicity against predominantly less negatively charged mammalian cell membranes^11,14^.

Experimental approaches to investigate peptide/membrane interactions mainly focus macroscopic-averaged insight on a mesoscopic scale. A detailed description of peptide/membrane interactions at the atomic scale may help to gain information on CAMPs disruption mechanisms, permitting the rational design of new derivatives and, therefore, the better therapeutic use of these novel antimicrobials. Molecular Dynamics (MD) simulations provide atomic-level insights and have been widely used to study the interaction of AMPs with membranes^15–18^. MD simulations were also utilized to study BP100’s structure and activity: Wang Y. *et al*.^17^ performed an 8 µs-long MD simulation of BP100 in DMPC bilayer and found that BP100 remained in the surface-bound-state inserting through its C-terminus. They also reported the unfolding of BP100 N-terminus (Lys1 and Lys2) after 1.5 µs, resulting in a tilt angle of 97° and 81% helicity^10,17^. In a study with Brownian Dynamics simulations with a coarse-grained peptide model, Alves *et al*.^18^ simulated BP100 confined in model membranes at 293 K for 1 µs each and reported the high stability of BP100 pre-folded alpha helix and a threefold decrease in its lateral diffusion in POPC: POPG membranes relative to that in POPC membrane^18^.

**Figure 1:**
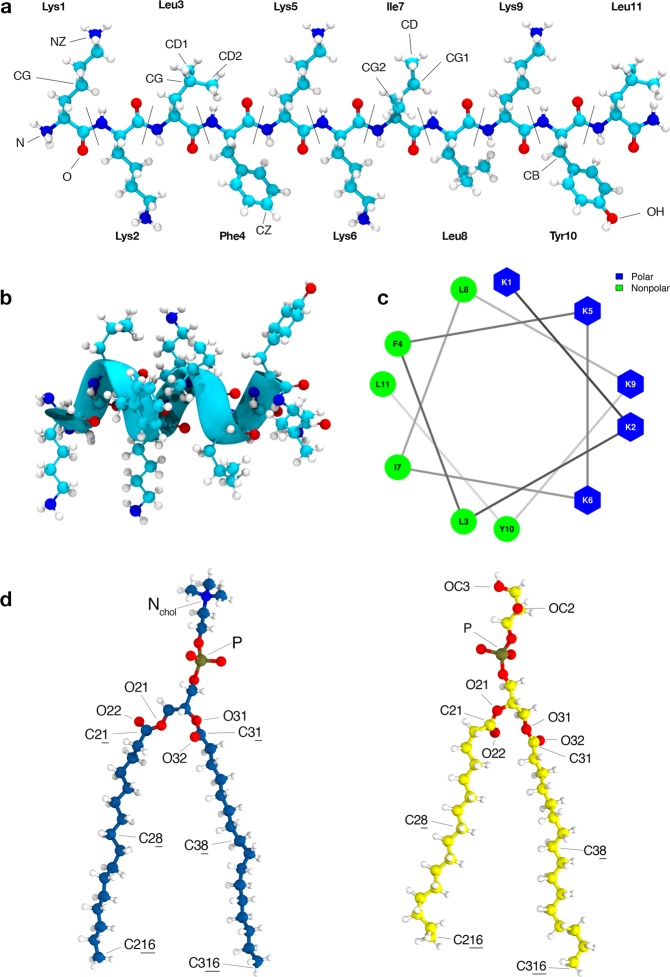
BP100 initial structures: random coil **L**-BP100 (**a**) and alpha-helical **α**-BP100 (**b**). Helical wheel projection (**c**), Lys5 and Ile7 were used as representative residues of polar and apolar moieties, respectively. In (**d**) DPPC (in blue) and DPPG (in yellow). Atom names are shown for peptide and lipids; they were used in our peptide-lipid pair analysis.

In this work we report simulations, in solution and membranes, extending for more than 25 µs of BP100 and membranes of 1,2-dipalmitoyl-sn-glycerol-3-phosphocholine (DPPC), 1,2-dipalmitoyl-sn-glycerol-3-phosphoglycerol (DPPG), and DPPC: DPPG mixtures (1:1). We systematically investigated the dependence of BP100 secondary structure on DPPG lipids which agrees with experimental findings^11^. We also analyzed the contribution of PG-containing lipid rafts on BP100 conformation and behavior. In most simulations containing DPPG lipids, we observed the occurrence of a dynamic transition of the peptide adsorbed at the membrane interface, where the apolar facet of the BP100 (Fig. 1c), initially exposed to water, rotates toward the membrane interior, leaving the polar facet of the peptide exposed to water. This process, defined here as peptide flip, could be part of CAMPs primary antimicrobial mechanism. Constraining four residues of the peptide into an alpha helix turn, we observed that the unconstrained regions of the peptide rapidly acquired an alpha-helix conformation in DPPG-containing membranes, suggesting that the formation of these small alpha-helical nuclei in peptide/membrane interactions may be the initial step for the peptide insertion into the hydrophobic core of the membranes.

## Methods

All simulations sets were assembled, carried out and analyzed using GROMACS 5.0.2 software package. For trajectories visual inspections and image rendering, VMD 1.9.2 software was used. Table 1 shows all simulation systems and its simulation time. The program used to analyze the hydration shell of the peptide using the Minimum Distance Distribution Function was developed in our group.

**Table 1:** Table containing all MD simulation sets carried out in this work. For bilayers, we used Slipids forcefield and ff99sb-ildn-NMR for BP100. All sets were simulated at 323 K (above DPPC and DPPG transition temperatures) and 1 bar. **L**-BP100 indicate constraint-free simulations using random coil BP100 as peptide starting configuration and **α**-BP100, as alpha helix. Res1–5, res4–8 and res7–11 indicate simulations with BP100 with permanent constraints on the referred residues.

Simulation sets				
	Simulations	Peptide/Lipid	Waters/Lipid	Time (ns)
Bilayers	DPPC	0/128	54	860
	DPPG		53	1100
	PCPG-R	0/[64/64]	54	1200
	PCPG-NR		52	1200
Peptide in water	**L**-BP100 in water	1/0	—	2000
	**α**-BP100 in water		—	2850
Peptide with bilayers	**L**-BP100 in DPPC	1/128	54	1700
	**α**-BP100 in DPPC		54	2000
	**L**-BP100 in DPPG	1/128	53	1700
	**α**-BP100 in DPPG		53	2000
	**L**-BP100 in PCPG-R	1/[64/64]	54	1600
	**α**-BP100 in PCPG-R		54	2000
	**L**-BP100 in PCPG-NR	1/[64/64]	52	1700
	**α**-BP100 in PCPG-NR		52	2000
Partially constrained peptide in DPPG	**α-**BP100 (res1–5) in DPPG	1/128	54	600
	**α-**BP100 (res4–8) in DPPG		54	600
	**α-**BP100 (res7–11) in DPPG		54	600

### Lipid bilayers

**Figure 2:**
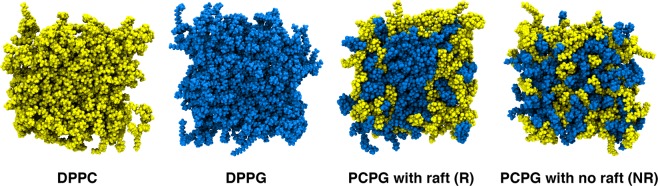
Initial membrane configurations membranes.

We used Slipids forcefield^19^ parameters were chosen to simulate DPPC, DPPG, and DPPC/DPPG mixed bilayers. 64-lipids-per-monolayer DPPC and DPPG pure membrane topologies were taken from the Slipids developer group website (http://www.fos.su.se/~sasha/Slipids/Downloads.html). Mixed bilayers of DPPC and DPPG containing 32 DPPCs and 32 DPPGs in each leaflet were constructed using the PACKMOL^20^ software. One membrane was composed of randomly distributed lipids (PCPG-NR) on both leaflets and the other, a 4 × 4 DPPG raft was placed in the center of both monolayers (PCPG-R) and the remaining 48 lipids were randomly distributed (Fig. 2).

TIP3P water molecules were used to solvate our systems, with an approximately 53 water/lipid ratio. Na^+^ ions (Aqvist^21^) were included to counter-balance charges of membranes containing DPPG.

All bilayer sets were minimized and equilibrated for 20 ps in a NVT ensemble and thereafter, in NPT conditions for 50 ns. After that, the bilayers were simulated for 860 ns (DPPC), 1100 ns (DPPG) and 1200 ns (for both PCPG-R and PCPG-NR).

The area per lipid and membrane thickness were calculated for all membranes and thNen compared with experimental data and those obtained by Slipids authors (Tables S1 and S2). The last trajectory frames of the membrane simulations were used to build our peptide-lipid systems.

### Peptide-in-solution and peptide-lipid systems

Following a study by Beauchamp *et al*.^22^, ff99sb-ildn-NMR force field was chosen to characterize BP100 in our modeling due to its good performance in simulating peptides and proteins structures in TIP3P water when compared with NMR measurements.

BP100 (H-Lys-Lys-Leu-Phe-Lys-Lys-Ile-Leu-Lys-Tyr-Leu-NH_2_) has an amidated C-terminal and a +6 charge at physiological pH, with all its Lysines and N-terminal amine protonated.

To study secondary structure behavior of BP100 both in solution and in bilayers, two initial BP100 conformations were used: linear BP100 (**L**-BP100) and alpha-helical BP100 (**α**-BP100) (Fig. 1 a,b). In the peptide/water system, one peptide (**α**-BP100 or L-BP100 initial conformation) were simulated in a cubic box with 3975 TIP3P water molecules for more than 1 microsecond with its +6 charges neutralized with 6 Cl^**−**^ ions (Dang^23^). Simulations containing peptides with membranes had a peptide/lipid ratio of 1/128 and the peptide was initially positioned in parallel orientation with the membrane surface approximately 2 nm away. For the **α**-BP100, additionally, we tested two parallel orientations, where the polar amino acids were facing the membrane and a random orientation. For the perpendicular orientation we tested two ways with either the C-terminal or N-terminal facing the interface. For all these initial conditions, we obtained the same final location and orientation of the peptide that will be discussed in the results section.

**Figure 3:**
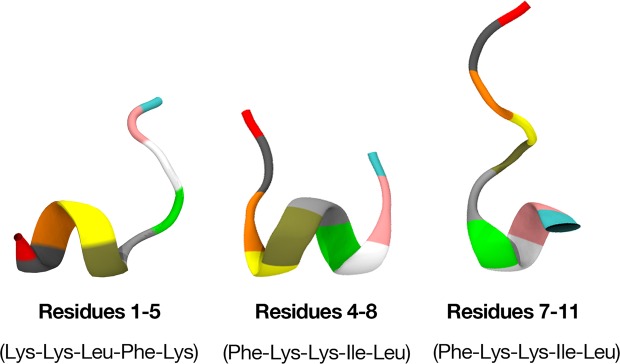
Starting structures for peptides with permanent constrained dihedral angles.

To investigate the influence of small core alpha-helical sequences in BP100 to its stability when interacting with bilayers, constrained simulations of BP100 in DPPG membranes were performed. The alpha-helix dihedral angles (ϕ = −60°; ψ = −40°) in 3 different regions were fixed: 1–5 (Lys-Lys-Leu-Phe-Lys), 4–8 (Phe-Lys-Lys-Ile-Leu) and 7–11 (Ile-Leu-Lys-Tyr-Leu) residues (Fig. 3). We maintained the constrained regions during the simulations.

Following the previous lipid membrane systems, energy-minimization, NVT, and NPT ensembles were used in the equilibration stage of the MD simulation.

### Simulation details

MD simulations were carried out with a 2 fs time-step with a Leap-Frog integrator^24^, in isobaric-isothermal (NPT) ensemble, at 323 K (50 °C) with temperature coupling treated separately for BP100, lipids, water and ions with the V-rescale thermostat^25^. Atmospheric pressure of 1 bar was kept with the Berendsen barostat^26^ and semi-isotropic pressure coupling was used for all systems.

All bonds were constrained with LINCS^27^ algorithm. Long-range electrostatic correction was treated by a Particle-mesh-Ewald method^28^ with a real-space cut-off of 1.5 nm. van der Waals interactions were truncated at 1.5 nm distance with a switch function from 1.4 nm.

### Simulation analyses

#### Lipid/peptide contacts

For the computation of lipid contacts with BP100 and pair frequency, we labeled some lipid(s) and BP100 atoms as “polar” or “apolar”, based on charges. Then, the radial distribution function (RDF) between each pair of apolar (or polar) atoms of the lipids/BP100 system were computed. The time window selected for computing these RDFs includes that of the membrane bound peptide 250 ns-before and 250 ns-after the peptide flip. The pairs with distinguishable RDF peaks and following minimum were used to analyze the number of polar and apolar contacts between lipids and peptide. A cutoff distance for each pair was defined from the position of the first minimum in the RDF curve, and a contact was defined as a pair of atoms at smaller distance than their corresponding cutoff distance. The simulations were analyzed and the total number of polar and apolar contacts as a function of time was obtained. Additionally, some polar and apolar pairs were analyzed individually.

#### Peptide hydration

The time evolution of the solvent shell surrounding the peptide in its α and L conformations in all simulated membranes was analyzed using the minimum distance distribution function (MDDF)^29^ between the peptide and the water molecules, as well as the average number of water molecules N(r), within a distance r = 0.5 nm, named here as N(0.5). For comparison the MDDF of BP100 in water was used to define the bulk water hydration shell at the same distance.

## Results

The 17 sets of simulations (Table 1) were run for more than 25 µs of simulation time, and each unconstrained peptide/membrane simulation had at least 1.6 µs of simulation. After comparing the simulated membrane properties with experimental and other computational studies (Tables S1 and S2), we systematically analyzed the peptide conformation in solution and on bilayers, peptide trajectory, and dynamics on membranes and the effect of BP100 on model bilayers. In the following sections, we describe our results.

### Secondary structure

#### Peptide in solution

**Figure 4:**
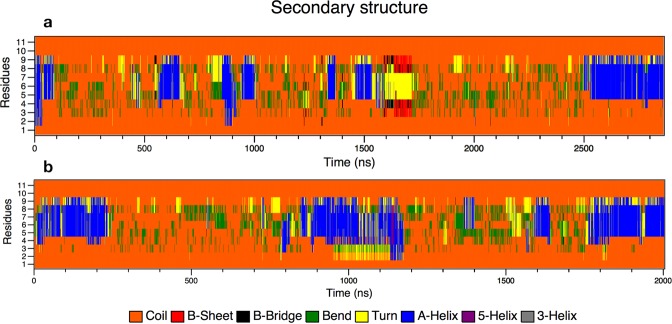
Secondary structure analyses graphs of **α**-BP100 (**a**) and **L**-BP100 (**b**) in solution. In both cases, the peptide shows predominance in random coil structure (orange) with occasional formation of 1 alpha helix turn (blue) in residues 5 to 9.

To study BP100 secondary structure in water, we did simulations for more than 2 µs with both α-BP100 and L-BP100 initial conformations. Figure 4 shows that BP100 conformation in solution was, predominantly, random coil. The peptide lost the initial alpha helix conformation in approximately 40 ns when starting with an alpha-helix configuration and occasionally formed an alpha-helix turn in the 5–9 residue region (Fig. 4a). For L-BP100 in solution, we observed a similar pattern (Fig. 4b). Circular Dichroism (CD) shows that BP100 is random in solution^10,11,30^, and our simulations reproduced these data.

#### Peptide in bilayers

**Figure 5:**
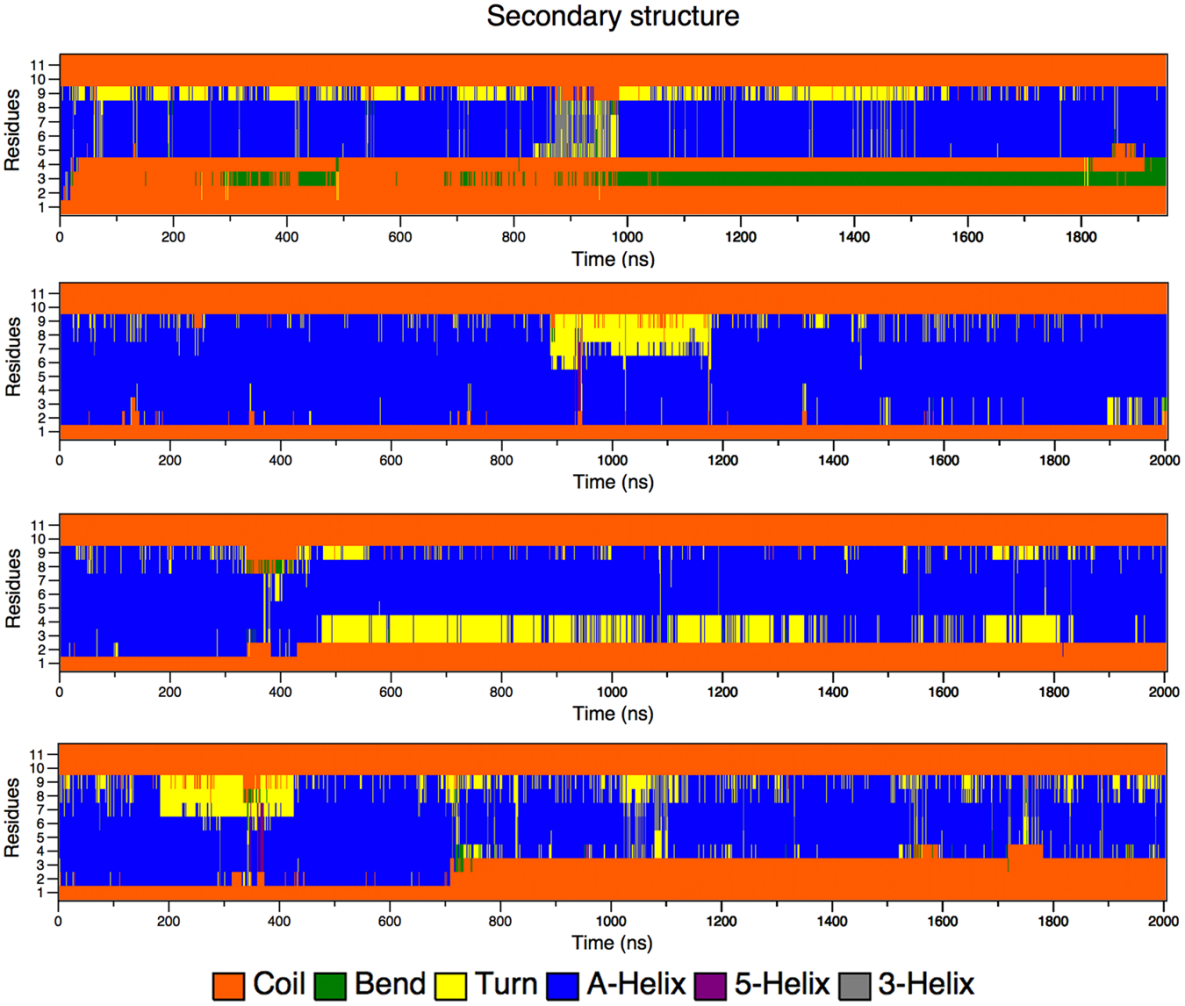
Secondary structure analyses of **α**-BP100 in DPPC (**a**), DPPG (**b**), PCPG-R (**c**) and PCPG-NR (**d**).

BP100 conformation, antimicrobial activity, and PG content are related^11^. We simulated α-BP100 and L-BP100 on DPPC, DPPG, PCPG-R, and PCPG-NR at 323 K, above DPPG and DPPC transition temperatures. In all cases, the peptide was rapidly adsorbed on the membranes and diffused laterally. Figure 5 shows that the BP100 alpha-helical conformation was proportional to PG content, as the alpha-helix percentage in DPPC is considerably smaller than that observed in DPPG-containing membranes. Alpha-helix formed in the 5 to 9 residues region (Lys-Lys-Ile-Leu-Lys) was highly stable in all simulations.

In DPPC, α-BP100 alpha-helix conformation reached a plateau at 38% helicity throughout the simulation, being relatively less stable than α-BP100 in bilayers containing DPPG, where a higher propensity of maintaining its conformation was observed. For pure DPPG (Fig. 5b) we found 73% (8 out of 11 residues), and for both PCPG (1:1) membranes we report 54% (6 out of 11) of helicity (Fig. 5c,d). No alpha-helix formation was detected in L-BP100 on bilayers simulations (Fig. S3). Therefore, the 1.8 µs simulation was not enough to present a spontaneous peptide folding.

The alpha-helix formed in the region of the residues 5 to 9 showed higher stability in all simulations with α-BP100 in bilayers (Fig. 5). This secondary structure pattern led us to suggest that the residues 5 to 9 are related to helix nucleation, in which the formation of this helix core could be the first step in the AMP actions.

**Figure 6:**
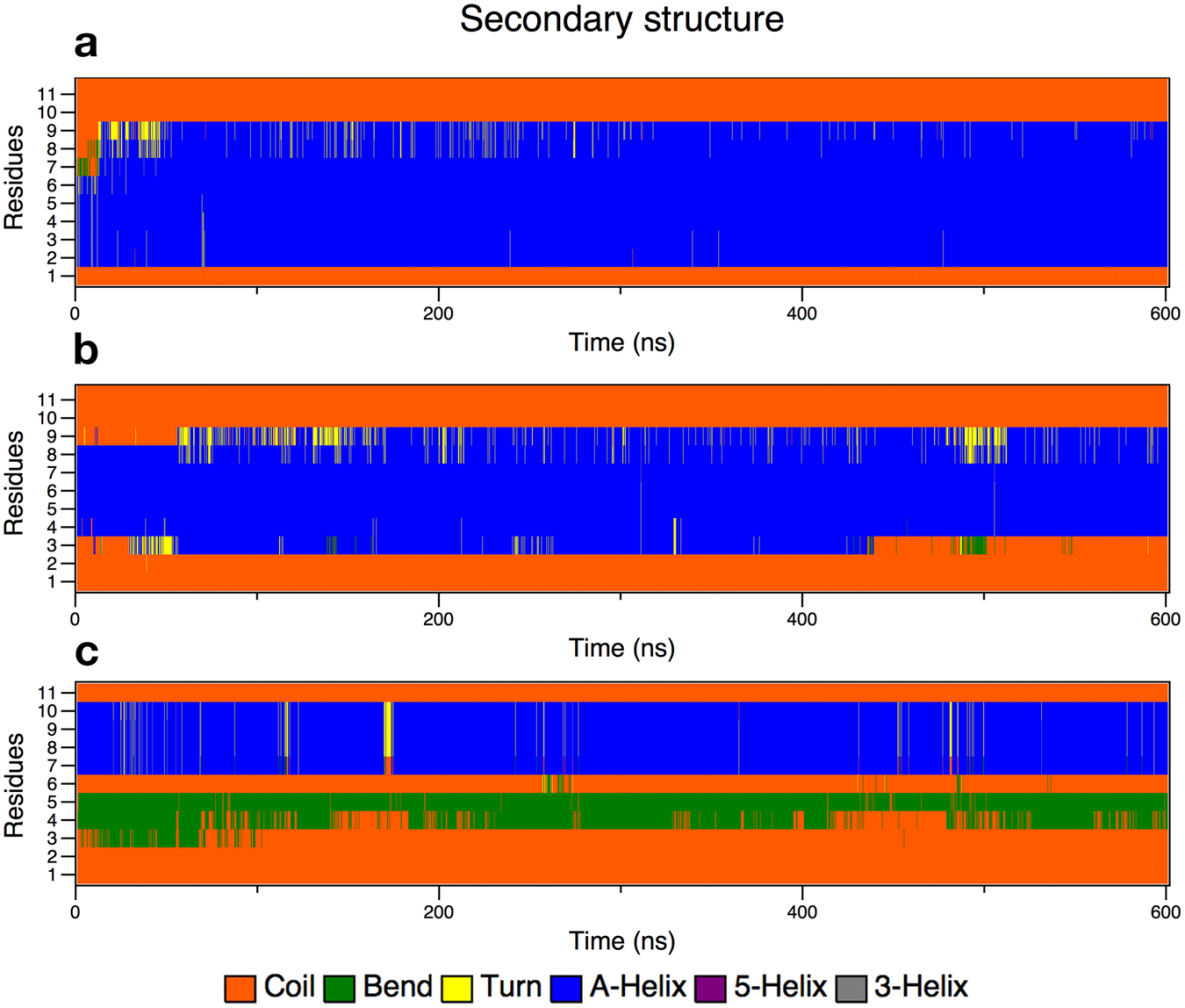
Secondary structure analyses graphs of BP100 in DPPG with one turn constraints on res1–5 (**a**), res 4–8 (**b**) and res7–11 (**c**).

Following this assumption, we tested three conformations with α-BP100 torsionally constrained in three regions: res1–5, res4–8, res7–11 (Fig. 3) and simulated with pure DPPG membranes. Figure 6 shows the evolution of the secondary structure in each simulation.

α-BP100 with constraints on residues 1–5 (Lys-Lys-Leu-Phe-Lys) acquired identical helicity (73%) to α-BP100 with no constraints at the same conditions (Fig. 5b), after 50 ns of simulation (Fig. 6a), with a helix spanning from Lys2 to Lys9 (Fig. 6a). Constraints on residues 4–8 (Phe-Lys-Lys-Ile-Leu) showed an overall 52% of helicity (Fig. 6b), with a helix spanning from Phe4 to Lys9 (Fig. 6b). Constraints on residues 7–11 (Ile-Leu-Lys-Tyr-Leu) did not affect the rest of the peptide conformation (Fig. 6c) in 600 ns of simulation.

### Peptide flip

**Figure 7:**
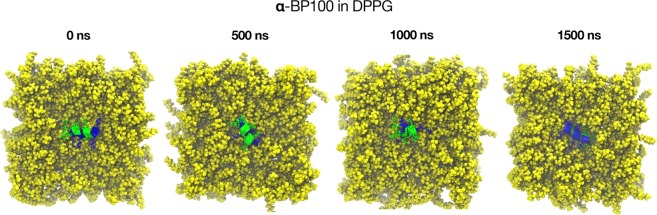
Peptide flip observed from above in **α**-BP100 in DPPG set after *circa* 1400 ns of simulation. In blue, polar facet and in green, the apolar facet. Peptide flip was also observed in **α**-BP100 in DPPC, **α**-BP100 in PCPG-R, and **α**-BP100 res(1–5) in DPPG simulations.

Simulation trajectories analyses (Fig. 7) revealed that the peptide in alpha-helix conformation initially adsorbed at the DPPG-containing membranes interface with its hydrophilic facet in contact with the membrane, leaving the hydrophobic facet exposed to water. We observed that the alpha-helix rotated, inserting the hydrophobic facet into the hydrophobic region of the membrane. We define this dynamic phenomenon, observed in our simulations, as peptide flip. Although this final peptide orientation is known^31–34^, no MD simulation on peptide flip has been reported.

**Figure 8:**
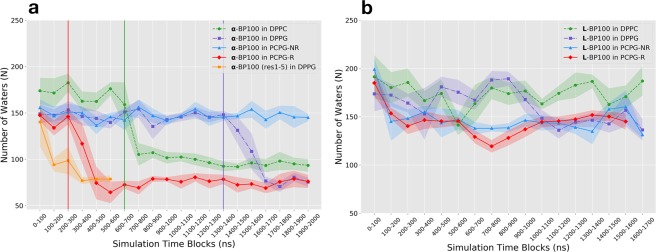
Average number of water molecules up to a distance of 0.5 nm, N(0.5), obtained from the minimum-distance distribution function (MDDF), between L-BP100 (**a**) and **α**-BP100 (**b**) and water molecules. Vertical lines indicate when peptide flip occurred.

The peptide flip caused dehydration (Fig. 8). When in contact with membranes, depending on the secondary structure of the peptide and the lipid nature, the hydration of the peptide was slightly different. However, when the peptide flip took place, its hydration was drastically altered.

In the peptide/membrane simulations with L-BP100 as a starting configuration (Fig. 8a), no significant change in the number of water molecules surrounding the peptide was observed in comparison the 150 obtained in the bulk aqueous solution. In the last 500 ns of simulation, N(0.5) was approximately 178 ± 11 in DPPC, 145 ± 7 in DPPG, 145 ± 13 in PCPG-NR, and 148 ± 3 in PCPG-R. No peptide flip was detected in the trajectories for those simulations. However, in the peptide/membrane simulations with α-BP100 as a starting configuration (Fig. 8b), N (0.5) lowered in DPPC (starting at ~700 ns), DPPG (~1500 ns) and PCPG-R (~390 ns). Alongside with dehydration, a peptide-flip was observed in DPPG and PCPG-R. For the **α-**BP100 in DPPC simulation, we observed a semi-flip, as the peptide alpha-helical conformation was only 38% (Fig. 5a), suggesting that the peptide overall polar/apolar segregation (Fig. 2c) was lost. The peptide flip transition time varied from 100 to 400 ns.

**Figure 9:**
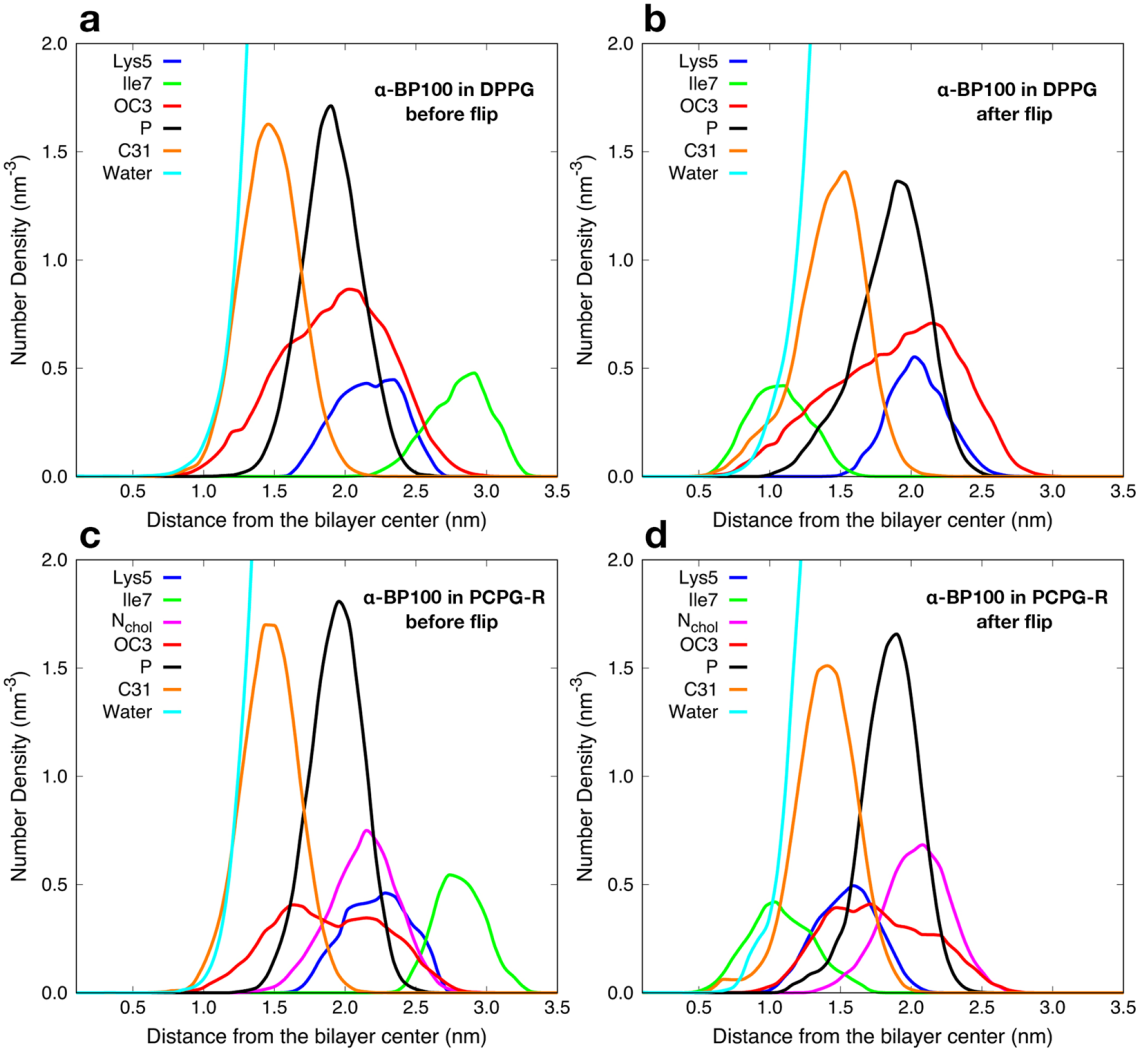
Peptide flip observed through number density analysis. (**a** and **b**) show respectively before and after the flip in **α**-BP100 in DPPG simulation; c) and d) demonstrate the flip for **α**-BP100 in PCPG-R simulation. Lys5 represents the polar side of BP100 and Ile7, the apolar facet (Fig. 2c). OC3 (DPPG head group further out glycerol Oxygen), N_chol_ (DPPC head group choline Nitrogen), P (Phosphorus) and C31 (first carbon from sn-1 acyl chain) are shown to display BP100 insertion into the bilayer after the flip.

During the peptide-flip, in the simulations of α-BP100 in DPPG and PCPG-R, the mean number N(0.5) was reduced from 147 ± 5 and 142 ± 7 to 76 ± 4 and 75 ± 4, respectively. These represent on average a reduction of approximately 48% in the number of water molecules in the hydration shell of α-BP100. In DPPC, we detected a smaller decrease in the hydration of α-BP100, around 45% (from 172 ± 8 to 94 ± 2), having around 20 water molecules more after the semi-flip. Figure 9 shows the density profile before and after peptide flip for α-BP100 in DPPG and PCPG-R. We chose residues Lys5 and Ile7 to illustrate the changes in the mean position of hydrophobic and hydrophilic residues with respect to the membrane due to the peptide flip. These residues are polar and apolar, respectively, and located opposite to each other when in an alpha-helix conformation (Fig. 2c). Trajectory visualization and density profile (Figs 7 and 9) clearly show the distinct orientation in BP100 hydrophobic facet before and after peptide flip. During the initial phase of approaching towards the membrane, BP100 polar side faces the membrane with its N-terminal residues (Lys1 and Lys2) interacting with the bilayer headgroups. After the flip, the apolar residues facet turns to the membrane, and simultaneously inserts into the bilayer, below the carbonyl carbons (C31) of the lipids (Fig. 9).

Peptide flip was not observed in α-BP100 in PCPG-NR, where DPPG lipids were initially scattered randomly. PG content might be related to the occurrence of the flip, but also, the PG content in the vicinity of BP100. Several studies suggest membrane-active peptides cause anionic lipid clustering, or subdomain formation, upon binding to bilayers containing neutral and charged phospholipids^35–37^. The same mechanism could be involved in BP100/bilayers interactions.

### Lipid clustering

**Figure 10:**
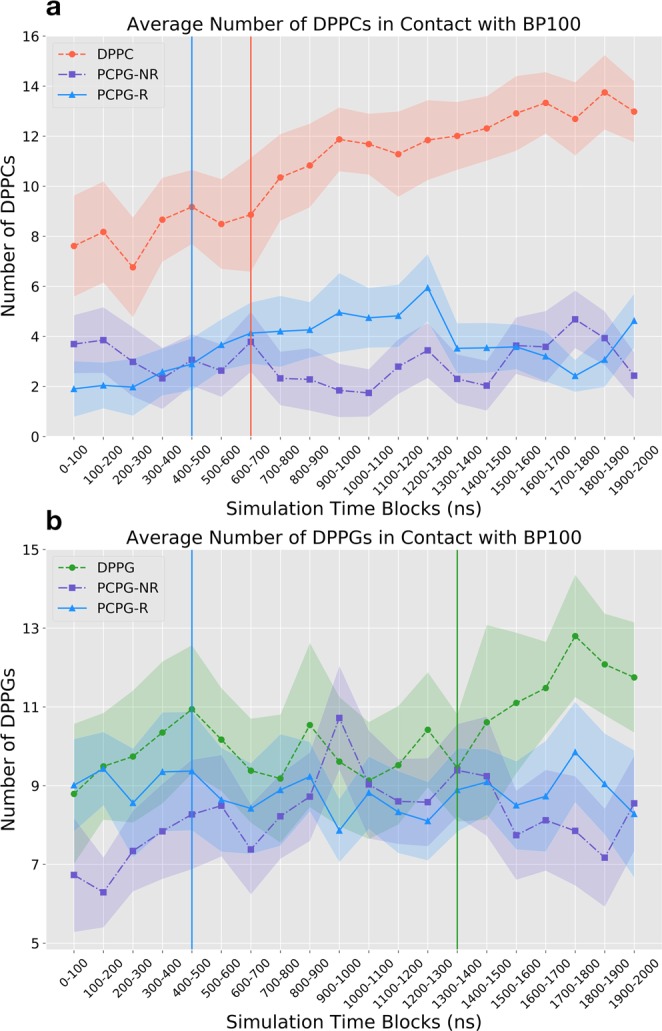
Average Number of DPPCs (**a**) and DPPGs (**b**) in contact with BP100 analyzed for all constraint-free simulations containing BP100 in an initially alpha helical conformation in DPPC, DPPG, PCPG-R and PCPG-NR membranes. Vertical lines represent the average time where peptide flip was observed. Standard deviation error bars are represented as fillings.

The distribution of the lipids in the vicinity of BP100 was evaluated by computing the total number of lipids in contact with α-BP100 as a function of time (Fig. S5), the time-averaged values (Table 2, Fig. 10), and the lipid raft size distribution (Fig. S6).

**Table 2:** Average number of lipids in contact with BP100 in simulations using **α**-BP100 as the peptide starting configuration, except **α**-BP100 (1–5), which had permanent constraints on residues 1 to 5. Standard deviations are shown in parenthesis.

Average Number of Lipids in Contact with BP100		
Simulation	Lipid in Contact	Average
**α**-BP100 in DPPC	DPPC	10.7 (±2.6)
**α**-BP100 in DPPG	DPPG	13.1 (±1.4)
**α**-BP100 (1–5) in DPPG	DPPG	13.5 (±1.9)
**α**-BP100 in PCPG-R	Total	12.3 (±1.7)
	DPPC	3.6 (±1.5)
	DPPG	8.7 (±1.2)
**α**-BP100 in PCPG-NR	Total	11.0 (±1.7)
	DPPC	2.7 (±1.3)
	DPPG	8.3 (±1.6)

α-BP100 in DPPC simulation had on average 10.7 peptide/lipid contacts (Table 2) and a steady increase in the number of contact lipids (Figs 10b and S5a). This increase is due to the peptide losing its alpha-helical conformation (Fig. 5a), expanding the available peptide interaction area with the membrane. Lipid raft size distribution also shows a broader distribution of DPPC raft size, supporting this observation (Fig. S6a). A semi-peptide flip was observed at ~700 ns of simulation and, concomitantly, while BP100 inserts into the bilayer as the density profile shows (Fig. S4a,b), the number of DPPCs in contact with BP100 increased (Fig. S5a).

α-BP100 in DPPG simulation shows on average 13.1 DPPGs in close contact with the peptide (Table 2). The number of DPPGs in contact rose after the flip, approximately in 1500 ns of simulation (Figs 10a and S5b) and concurrently, BP100 inserted deeply into the membrane (Fig. 7a,b).

Constrained α-BP100 (res1–5) in DPPG, which showed identical alpha-helix content with α-BP100 in DPPG (73%, Figs 5b and 6a), not only exhibits peptide flip in approximately 300 ns of simulation but also has, on average, 13.5 DPPGs in contact with BP100 (Table 2). In the simulation with α-BP100 in PCPG-NR, the number of DPPG in contact with the peptide showed a slight increase (Fig. 10b), reaching the average value observed in the simulation with PCPG-R (Table 2). Also, lipid raft size probability analysis (Fig. S6d) showed a narrower peak distribution for DPPGs in contact with BP100 in PCPG-R system, compared to PCPG-NR (Fig. S6e), indicating that DPPG lipid raft size was stable. Peptide flip was observed in the PCPG-R set after ~390 ns of simulation and no significant variation of the number of DPPG in contact with peptide was observed.

### Polar and apolar interaction analysis

**Figure 11:**
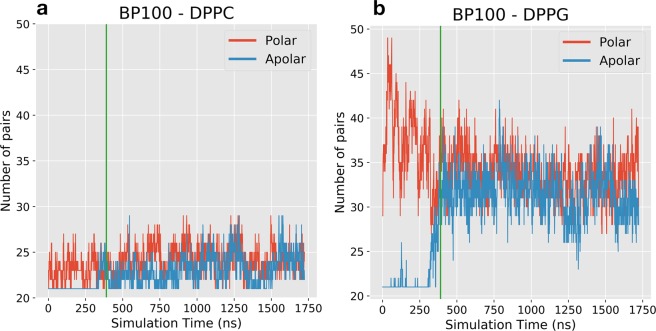
BP100 pair occurrence with DPPC (**a**) and DPPG (**b**) for **α**-BP100 in PCPG-R simulation. Graphs show the sum of the ten most frequent apolar/polar pairs during the simulation. Pairs were calculated using the monolayer facing the peptide. Green vertical lines indicate when peptide flip occurred (*circa* 390 ns).

Peptide contact analysis revealed the increase of lipid/peptide contacts after peptide flip and DPPG clustering on PCPG membranes. We analyzed the nature of the intermolecular interactions that determine peptide-membrane interactions throughout the simulations. We calculated the number of polar and apolar pair contacts between peptide atoms and membrane atoms (Figs S7–S13). Using α-BP100 in PCPG-R system as an example, Fig. 11 shows the sum of the 10 most frequent apolar/polar pairs between BP100 with DPPC and DPPG.

The total number of pairs, regardless if it is polar or apolar, between BP100 and DPPG is higher than that with DPPC (Fig. 11), which directly reflects on the number of lipids in contact with BP100 (Fig. 10). During the initial approach of the peptide towards the bilayer, polar interactions between charged Lysines and DPPG headgroup dominate the interaction (Figs 11b, S9, S10, S12 and S13). The primary interaction was between positively charged (+1) Lysine side chain amino group (NZ) and DPPG head phosphoglycerol group oxygens (OC2, OC3), even after peptide flip (Figs S9, S10, S12 and S13).

Before the peptide flip, the number of polar contacts is higher than the apolar contacts for DPPG/BP100. As the peptide starts to flip, the number of apolar contacts increase, while the polar contacts decrease. After the peptide flip is completed, apolar contacts are as numerous as the polar contacts (Fig. 11a,b and S7–10,13). Also, after the flip, apolar contacts between BP100 and DPPG were more frequent than with DPPC (Fig. 11b). The higher peptide/DPPG apolar contact frequency after the flip is also observed for other simulations with peptide flip (Figs S7–10, 13).

## Discussion

### Peptide secondary structure

CD spectra of BP100 in solution and POPC LUVs show no secondary structure^10,11,30^ Our simulations with BP100 in solution, and DPPC membrane exhibited the same pattern. α-BP100 and L-BP100 in solution showed similar behavior, with a prevalence of random conformation (Fig. 4). In a related MD study of AMPs interacting with membranes^17^, an 8 µs-long all-atom MD simulation of BP100 in DMPC at 35◽ the authors reported a full helix length during 1.5 µs and a subsequent unfolding event of Lysines at positions 1 and 2, resulting in 81% of helicity on a pure zwitterionic bilayer. However, as reported for other simulated peptides in the same work, the forcefield used could result in an over-helical artifact^17^. A Brownian Dynamics simulation study of BP100 confined inside an implicit POPC membrane reports a stable alpha-helix^18^. However, no experimental data confirms that BP100 diffuses freely in the membrane hydrophobic space and no secondary structure formation is reported for pure POPC vesicles^11^.

In our simulations of α-BP100 and L-BP100 in DPPC membrane, the peptide quickly approached the membrane and remained on the surface during all simulation. Peptide-lipid pair analysis (Fig. S7) showed that cationic Lysine residues interacted mainly with phosphate groups of the DPPC bilayer, evidencing that electrostatic interactions were present.

In the case of L-BP100 in DPPC, no alpha-helical structure formation was observed (Fig. S3), and for α-BP100, the peptide alpha-helix conformation was mostly lost, although one helix turn from residues 5 to 9 remained (Fig. 5A). The minimum alpha-helix length detected by CD spectroscopy is proposed to encompass seven to eleven residues^38^, hence the alpha-helix turn in residues 5–9 might not be detected by CD. Alternatively, this could be an over-helical forcefield artifact^22^, but it also indicates the high stability of the alpha-helix formed by the (5–9) Lys-Lys-Ile-Leu-Lys sequence. This stability leads us to conjecture that BP100 alpha-helix formation could be initiated through a core sequence, a nascent helix^39^. The negatively charged membrane interface could promote the initial peptide folding. To support this, we performed three 600 ns-long simulations with constrained alpha helices of BP100 on three regions: residues 1 to 5 (Lys-Lys-Leu-Phe-Lys), 4 to 8 (Phe-Lys-Lys-Ile-Leu) and 7 to 11 (Ile-Leu-Lys-Tyr-Leu) in pure DPPG bilayers. The secondary structure analysis of the constrained BP100 simulations yielded different outcomes. Constraint on residues 1 to 5 rapidly induced almost full alpha-helix content from Lys2 to Lys9 (Fig. 6A), reaching 73% of helicity, similar to α-BP100 in DPPG without constraints (Fig. 5B). Constraint on residues 4 to 8 also showed a helix formation tendency (overall 63% helicity, 5 out of 11 residues, from Phe4 to Lys9). In sharp contrast, constraints on residues 7 to 11 (36% of helicity, 4 out of 11 residues, from Lys7 to Tyr10) had no effects on the secondary structure formation of BP100 during 600 ns simulation (Fig. 6C), indicating that this region has little influence on initial peptide folding.

Pace and Scholtz^40^, based on experimental studies, proposed a helix propensity scale as Δ(ΔG) values relative to Alanine set to zero. On the Pace and Scholtz scale, the 5–9 region has the highest helix promoting residues (overall helix propensity of 1.4 kcal/mol). We find 1.53 kcal/mol for residues 1 to 5 (Lys-Lys-Leu-Phe-Lys), 1.68 kcal/mol for residues 4 to 8 (Phe-Lys-Lys-Ile-Leu), and 1.62 kcal/mol for residues 7–11 (Ile-Leu-Lys-Tyr-Leu). Our secondary structure data indicates that BP100 nascent helix could be formed either in the region between residues 1 to 5 or between residues 5 to 9. We favor the latter due to the presence of positively charged Lysine residues in the N-terminal side (Lys1 and Lys2) which could produce an energetically unfavorable barrier to initial peptide folding. The 5–9 region shows high stability in all constraint-free α-BP100/membranes simulations and has the overall lowest energy barrier in the helix propensity scale^40^.

DPPG seems to have a helix-stabilizing effect on BP100. All the simulations with α-BP100 in DPPG-containing-membranes showed high alpha-helical content, including the region of residues 5 to 9. Our helicity results from the simulations with α-BP100 in pure DPPG (73%) and in PCPG (50:50) mixed bilayers (54%) are remarkably close to those reported in experimental studies: 54% in POPC:POPG (50:50)^11^ and 61% in DMPC/DMPG (75:25) LUVs^10^, and maximum observable alpha-helix spectrum for BP100 in pure POPG vesicles^11^ proving that BP100 alpha-helical structure is dependent on PG content^11^.

For L-BP100 simulations with DPPG, PCPG-R, and PCPG-NR, no secondary structure formation was detected (Fig. S3), with the peptide being in a random conformation. Non-biased random peptide simulations require much longer simulation time to sample the peptide conformational potential landscape or might dwell in several local minimum-energy states.

### Peptide flip

BP100 is too short to span the bilayer thickness, especially in the alpha-helical conformation, and thus single molecule pore formation is an unlikely scenario. NMR data of BP100 in DMPC: DMPG (3:1)^10^ shows that upon binding to negatively charged membranes, alpha-helical BP100 positions parallel to the membrane as in a surface-bound orientation, and has an azimuthal rotation angle close to 160°, with Lysine residues facing the solvent. This last arrangement is commonly observed with other CAMPs^30–33^.

Peptide/membrane simulations with initially alpha-helical BP100 had its polar moiety facing the membrane, for being the most probable scenario in a physical environment. In the α-BP100 in DPPG (Fig. 7), α-BP100 in DPPC, α-BP100 in PCPG-R and α-BP100 (res1–5) in DPPG simulations trajectories, we observed the binding of BP100 from bulk solution to the membranes with its Lysines residues pointing towards the bilayer, and then, the peptide rotated, leaving its apolar facet facing the interior of the bilayer. We termed this transition peptide flip. Although BP100 surface-bound state is reported^12,17^, no previous peptide/membrane MD simulation work has reported a peptide flip transition.

Peptide flip brings significant outcomes regarding BP100 localization in the membrane, in peptide/membrane interaction and the peptide hydration, possibly being a crucial step of carpet mechanism for BP100 and other CAMPs. Through the density profile, we observed deep penetration of the peptide into the membrane through its apolar residues with larger side-chains, after the flip (Fig. 7). BP100 hydration analysis also shows a decrease in the overall peptide hydration after the flip (Fig. 8). Apolar/polar pair interaction analysis (Fig. 11) revealed that after the flip, the number of contacts between apolar residues from the lipids and the peptide increases significantly, while the number of contacts between polar groups of the lipids and the peptides slightly decreases. In other words, peptide flip intensifies peptide binding to membranes burying the apolar residues of the peptide deeper into the membrane core.

We investigated what factors could lead to the occurrence of BP100 peptide flip. Membrane composition appears to have a significant role. For BP100 alpha-helical structure formation is favored in negatively charged membranes^8^. In our simulations, BP100 entirely flipped in DPPG enriched regions of the membrane. Peptide flip was detected in α-BP100 in DPPG, α-BP100 (res1–5) in DPPG and α-BP100 in PCPG-R systems.

No peptide flip was detected in the other constrained BP100 simulations (α-BP100 (res4–8) in DPPG and α-BP100 (res7–11) in DPPG). However, it is hasty to affirm that it is unlikely to expect peptide flip to happen in those systems with 600 ns of simulation time. For instance, peptide flip was observed after 1.4 µs of simulation in α-BP100 in DPPG.

Hydration analysis (Fig. 8b) and density profile (Fig. S4A,B) for α-BP100 in DPPC show also that BP100 flipped. However, the alpha-helix secondary structure is mostly lost (Fig. 5a). For this reason, BP100 amphiphilic apolar-polar facet separation (Fig. 2c) is lost.

Additionally, the apolar moiety of the peptide could have a crucial role in peptide flip. Hydration analysis (Fig. 8) suggests peptide flip results in peptide dehydration and concomitantly enhances peptide/lipid interaction (Fig. 11), increasing specially apolar contacts (Fig. 11). Similar behavior was observed in a study of the interaction of micelles with polyatomic counterions with apolar and polar moieties. Experimental results and MD simulations showed that counterions with the same apolar groups but different polar groups resulted in similar ionic affinities for dodecyltrimethylammonium micelles, and the simulations showed that counterion adsorption to the micelles was related with the dehydration of the apolar group of the counterions rather than by the polar group^41^.

These factors could help to explain BP100 selectivity towards negatively charged bacterial membranes and low cytotoxicity. Results of leakage from the inner compartment of LUVs upon addition of BP100 showed that BP100 leakage ability is proportional to PG content while the peptide has small effects on zwitterionic PC vesicles, observed through studies of zeta potential and vesicle leakage^11^. BP100 does not perturb PC bilayers even at high peptide/lipid ratio and causes membrane thinning on mixed DMPC/DMPG bilayers where a stronger effect appears in anionic DMPG lipids compared to zwitterionic DMPC lipids^12^.

In zwitterionic-lipid-enriched mammalian membranes, BP100 would bind to membranes through electrostatic interactions and the absence of a helix-promoting environment would prevent helix formation and even with peptide flip, as in our α-BP100 in DPPC simulation, it could lead to mild membrane thinning^11,12^. On the other hand, bacterial membranes with high content of anionic lipids on the outer part of the membrane, could promote peptide binding, alpha-helix nucleation, and propagation, leading to peptide flip and more pronounced membrane thinning, which is consistent with our observations from α-BP100 in DPPG, α-BP100 in PCPG-R and α-BP100 (res1–5) in DPPG sets. Although we could not detect membrane thinning in our simulations (Table S2), it should be noted that our simulations had only one peptide on 128-lipid-bilayers. A more pronounced effect on the membrane might be observed by increasing the number of peptides in the simulations.

### DPPG subdomain formation

Lipid clustering, or subdomain formation, induced by CAMPs is well established^5,14,35–37,42–45^. Through electrostatic interactions, CAMPs clusters anionic phosphatidylglycerol (PG) or cardiolipin (CL) lipids on the outer membrane of bacteria, inducing phase separation and phase boundary defects between lipid domains and the rest of the membrane, increasing membrane permeability^36^. Epand *et al*. first proposed it^45^, in a study with oligomers of lysine (OAK). Upon the addition of OAK in 1-palmitoyl-2-oleoyl phosphatidylethanolamine (POPE) and tetraoleoyl cardiolipin (TOCL) mixed vesicles, transition temperature shift was observed through differential scanning calorimetry, indicating lipid phase separation^45^. Lipid phase separation was later observed for other cAMPs and cell-penetrating-peptides^37,45,46^. Although not so pronounced, DSC also detected BP100 ability to promote lipid phase separation on POPE/TOCL (75:25) vesicles^37^.

Lipid phase separation promoted by CAMPs has also been observed in computational modeling studies. In a coarse-grained MD simulation study^35^, lipid cluster promotion was seen in POPE/POPG (70:30) membranes by Ltc1, a linear alpha-helical cAMP with overall +10 charge. Simulations showed Ltc1 induced POPG domains in small (256 lipids) and large membranes (512 or 2048 lipids). Also, due to peptide-POPG charge neutralization, Ltc1-induced anionic domains seem to facilitate peptide oligomerization, enhancing peptide activity on bacterial model membranes^35^.

Likewise, our simulations suggest that BP100 promotes PG aggregation. Two DPPC: DPPG (50:50) mixed bilayers were designed to explore lipid aggregation by the peptide. One membrane was composed of randomly mixed (PCPG-NR) and in the other, a 4 × 4 DPPG raft (PCPG-R) was positioned in the middle of both monolayers (see Methods 1 and Fig. 1). BP100 was able to cluster DPPGs in PCPG-NR, which can be observed by the amount of DPPG in contact with BP100 (Table 2). From a total of 11 lipids in the closer contact with BP100, 8.3 in average are DPPG corresponding to 75% of the lipid in the vicinity of the peptide. Additionally, this DPPG clustering can also be observed in lipid raft size distribution (Fig. S6E), reaching similar values of DPPG/peptide contact in α-BP100 in PCPG-R simulation (Fig. 10) and DPPG lipid raft size probability (Fig. S6D). These data suggest that even a short-lengthened CAMP as BP100 can promote anionic lipid clustering and also maintain it, supporting the experimental findings^37^.

BP100 also adsorbs on pure zwitterionic PC membranes and promotes DPPC clustering as observed in Figs S5A and S6A. However, possibly due to the presence of positively charged choline headgroups (Fig. 1d - left), secondary structure is lost (Fig. 5a) and the peptide turns into random coil, resulting in an increase of available interaction surface area with the DPPC headgroups, which is reflected in a broad distribution in lipid raft size distribution (Fig. S5A). Polar/Apolar pair analysis for α-BP100 in PCPG-R (Fig. 11) helps to explain lipid aggregation. We computed the ten most frequent apolar and polar pairs between peptide and lipids. The number of polar pairs between the peptide and DPPG is higher than those with DPPC and is maintained even after the flip (~390 ns). As expected, +1 charged amino group (NZ) from Lysines side chains are the main participants in polar pairings (Figs S7–S13), interacting with DPPG headgroup glycerol (OC2, OC3, Fig. 1d - right), phosphate group (P), and carbonyl groups (C21, C31, O21, O22, O31, O32) from chains sn1 and sn2 (Figs S9, S10, S12, S13).

Peptide flip seems to contribute to lipid clustering. Polar/apolar pairs analysis (Fig. 11) and pair frequency (Figs S8, S9) for the previous simulation, reveals that apolar pairs between BP100 and both lipids rise after peptide flip occurs (~390 ns), intensifying peptide-membrane interaction. The same phenomena were observed for other simulations with peptide flip (Figs S7, S10 and S13). As long as peptide flip had occurred, apolar residue side chains (from Leu3, Phe4, Ile7, Leu8 and Tyr10, and Leu11) were in close contact mainly with groups of the lipid tails (Figs S7–S10, S13).

The mechanisms of bacterial toxicity via lipid clustering induced by CAMPs still has to be investigated. Moreover, possibly more than one mechanism could be involved in the process of bacterial killing. Evidence suggest lipid clustering by CAMPs could lead to negative charge concentration on membranes, promoting CAMPs attachment, leading to pore formation^5^. Anionic lipid clustering would have surrounding interfaces with the rest of the membrane which could lead to phase boundary defects, leading to slow leakage of inner cell content^32^. Also, CAMPs and cell-penetrating peptides could induce negative membrane curvature, enabling the internalization of peptides into cells, leading to intracellular killing mechanisms. The formation of lipid clusters patches would also lead to anomalies in membrane protein function, membrane fluidity, and membrane polarization. In a recent study^5^ with cWFW, a cyclic antimicrobial peptide, it was shown *in vitro* and *in vivo* that cWFW promotes lipid phase separation, a sharp decrease in membrane fluidity. The significant phase separation appears to cause disorganization of membrane proteins, inhibiting cell wall synthesis and inducing autolysis.

BP100 mode of action on membranes is dependent on peptide/lipid ratio^11,47^. As our studies comprised of low peptide/lipid concentration (1/128), we suggest that at low peptide/lipid concentrations, BP100 causes lipid aggregation on negatively charged membranes, leading to phase boundary defects between negatively charged lipid rafts and the rest of the membrane. Through this, membrane permeability would increase, leading to slow leakage of the cell inner content.

## Supplementary information

Supplementary Material

## Data Availability

The datasets generated during and/or analysed during the current study are available from the corresponding author on reasonable request.
