# Supplementary material for: Binding and Flip as Initial Steps for BP-100 Antimicrobial Actions
Source: Sci Rep. 2019 Jun 13;9:8622. doi: 10.1038/s41598-019-45075-5 (PMC6565725; doi:10.1038/s41598-019-45075-5)
Supplement: Supplementary file 1 — Supplementary Material [file 41598_2019_45075_MOESM1_ESM.pdf]

Supplementary Material

BINDING AND FLIP AS INITIAL STEPS FOR BP-100 ANTIMICROBIAL ACTIONS

Peter Park[1], Leandro R. Franco[2], Hernan Chaimovich[1,#], Kaline Coutinho[2], Iolanda M. Cuccovia[1,*], Filipe S. Lima[3,*]

* Correspondence to hchaimo@usp.br, filipe.lima@usp.br and imcuccov@iq.usp.br

[1] Departamento de Bioquímica, Instituto de Química, Universidade de São Paulo, São Paulo, Brazil;

[2] Departamento de Física Geral, Instituto de Física, Universidade de São Paulo, São Paulo, Brazil;

[3] Departamento de Química Fundamental, Centro de Ciências Exatas e da Natureza, Universidade Federal de Pernambuco, Recife, Brazil.

Contents:

- Experimental and theoretical area per lipid and membrane thickness values (S1, S2)
- Secondary structure analysis for random-BP100 in bilayers (S3)
- Number of lipids in contact with BP100 (S4)
- Lipid raft size probability (S5)
- Pair occurrence frequency between BP100 (S7-S13)
- MDDF between BP100 and the water molecules in solution (S14)
- References

Area per Lipid (Å²/Lipid)				
	Ensemble	Our Simulation	Experimental	Other simulations
Pure Bilayers	DPPC	61.7 (±1.0)	63.3[1]<br>67.2[2]<br>71.2[3]<br>62 (±1.3)[4]<br>63.1 (±1.3)[5]	62.6 (±0.5)[6]<br>61.8[7]<br>62.3 (±1.1)[8]
	DPPG	63.2 (±1.3)	67.0[9]	64.5 (±0.4)[10]
	PCPG-R	60.7 (±1.2)	-	-
	PCPG-NR	60.6 (±1.2)	-	-
Bilayers + Peptide	**L**-BP100 in DPPC	62.1 (±1.1)		
	**α**-BP100 in DPPC	62.3 (±1.2)		
	**L**-BP100 in DPPG	62.5 (±1.4)		
	**α**-BP100 in DPPG	62.3 (±1.3)		
	**L**-BP100 in PCPG-R	60.4 (±1.2)		
	**α**-BP100 in PCPG-R	60.8 (±1.3)		
	**L**-BP100 in PCPG-NR	60.7 (±1.2)		
	**α**-BP100 in PCPG-NR	60.1 (±1.2)		

Table S1) Comparison of area per lipid between experimental data and those obtained from our simulations. The average A_L was obtained by dividing the average xy area of the simulation by the number of phospholipids in a monolayer (64). No area per lipid data was found for PCPG (50:50) membrane experimental data.

Membrane Thickness (Å)				
	**Ensemble**	**Our simulation**	**Experimental**	**Other Simulations**
Pure Bilayers	DPPC	38.1 (± 0.6)	34.2[3]<br>39.0[5]<br>38.0[11]	37.7[6]<br>39.8[7]
	DPPG	37.0 (± 0.9)	35.5[9]	34.9[10]
	PCPG-R	38.3 (±0.6)		
	PCPG-NR	38.7 (± 0.8)		
Bilayers + Peptide	**L**-BP100 in DPPC	38.0 (±0.7)		
	**α**-BP100 in DPPC	38.1 (±0.6)		
	**L**-BP100 in DPPG	37.4 (±0.9)		
	**α**-BP100 in DPPG	37.7 (±0.8)		
	**L**-BP100 in PCPG-R	38.8 (±0.8)		
	**α**-BP100 in PCPG-R	39.1 (±0.9)		
	**L**-BP100 in PCPG-NR	38.6 (±0.8)		
	**α**-BP100 in PCPG-NR	39.1 (±0.9)		

Table S2) Comparison of membrane thickness between experimental data and those obtained from our simulations. Membrane thickness was calculated by averaging the distance between phosphorus atoms of each leaflet. No membrane thickness values for PCPG (50:50) membranes were found.

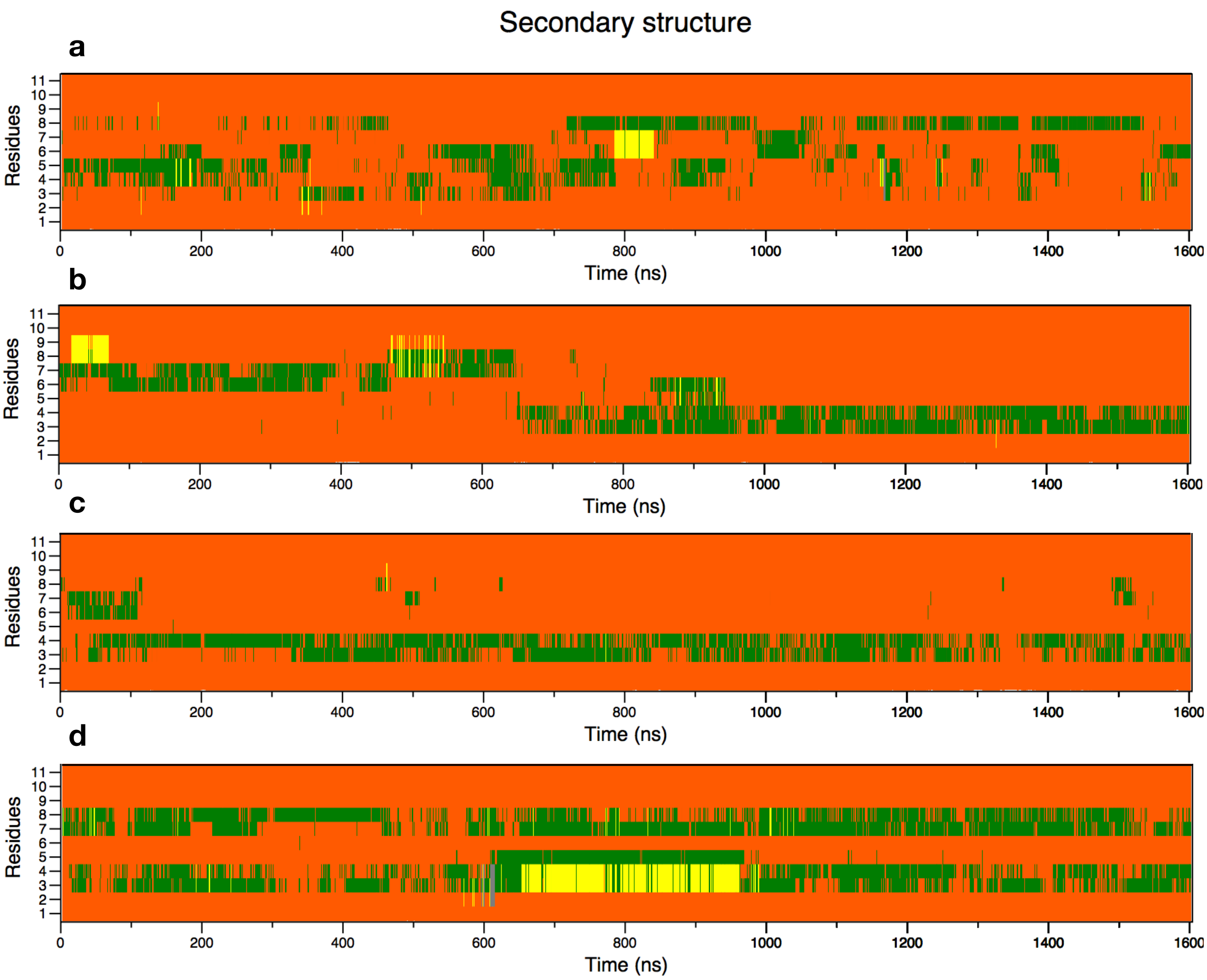

Figure S3: Secondary structure analyses graphs of **L**-BP100 in DPPC (a), DPPG (b), PCPG with raft (c) and PCPG without raft (d).

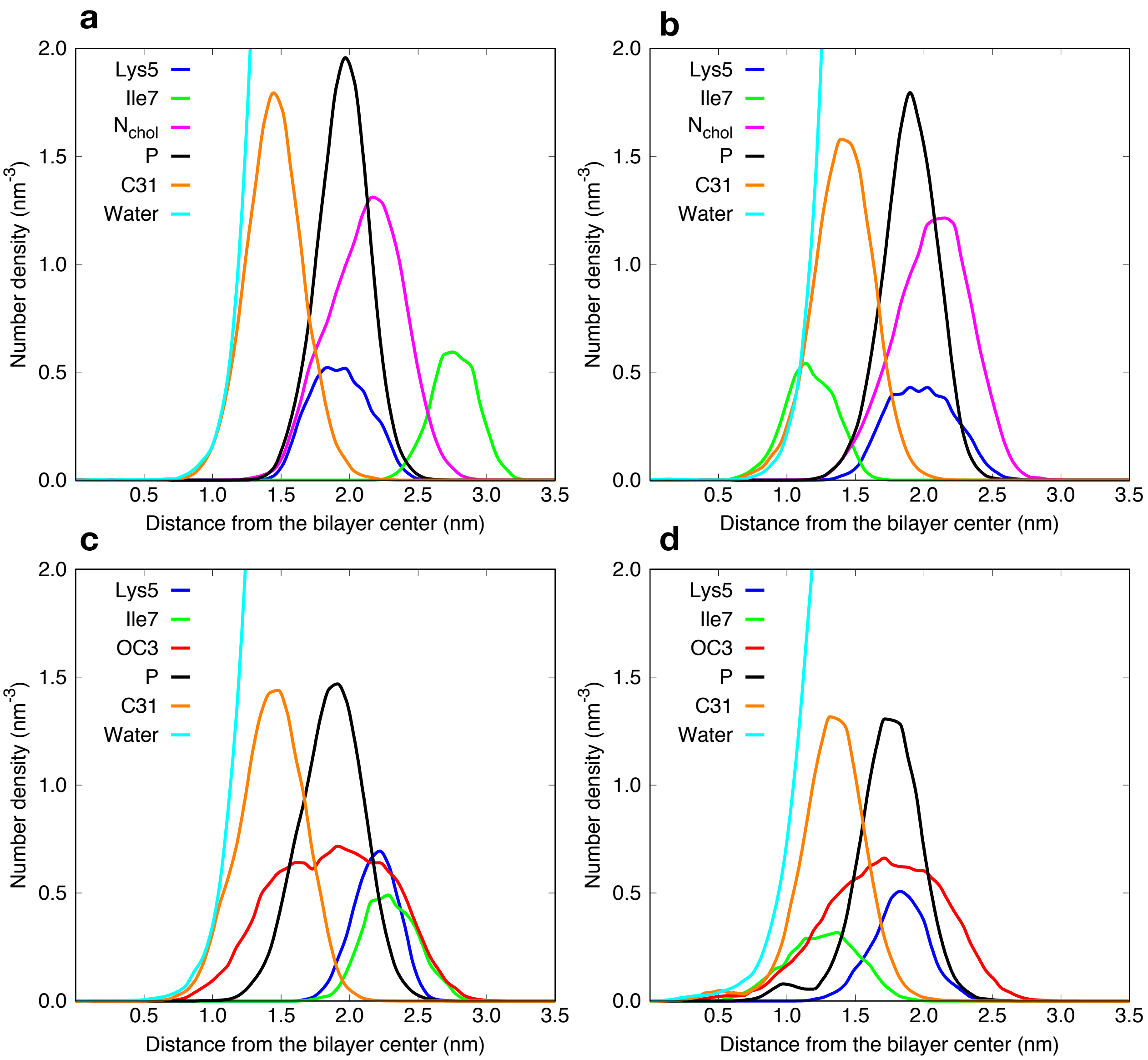

Figure S4) Peptide flip illustrated through number density graphs. a) and b) show respectively before and after peptide for **α**-BP100 in DPPC; c) and d) shows the peptide transition for **α**-BP100 (res1-5) in DPPG simulation. Deeper peptide insertion into the membrane happens after peptide flip. Concomitantly to peptide insertion, water infiltration was also noticeable.

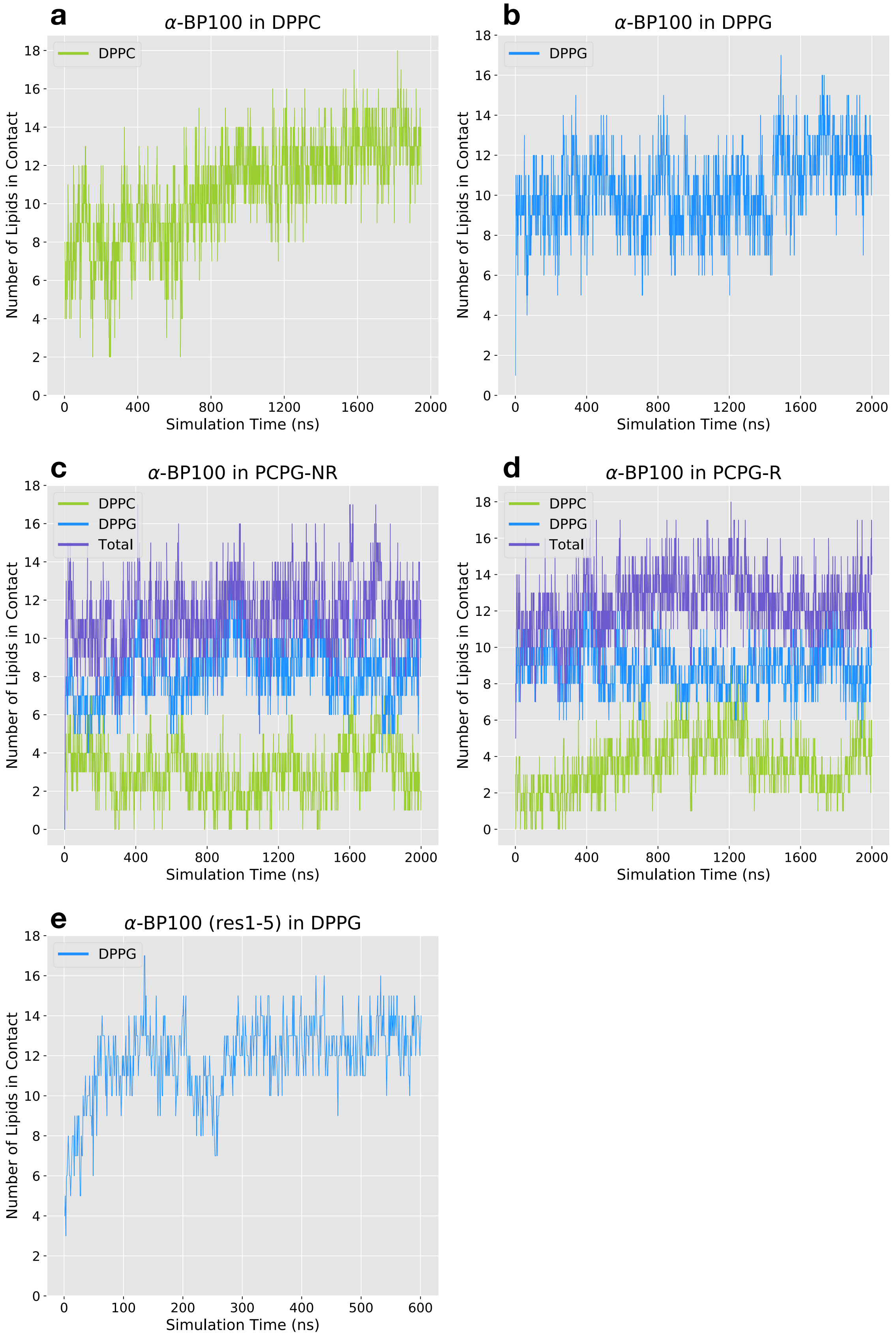

Figure S5) Total Number of Lipids in Contact with BP100 as a function of time analyzed for constraint-free simulations containing BP100 in an initially alpha helical conformation.

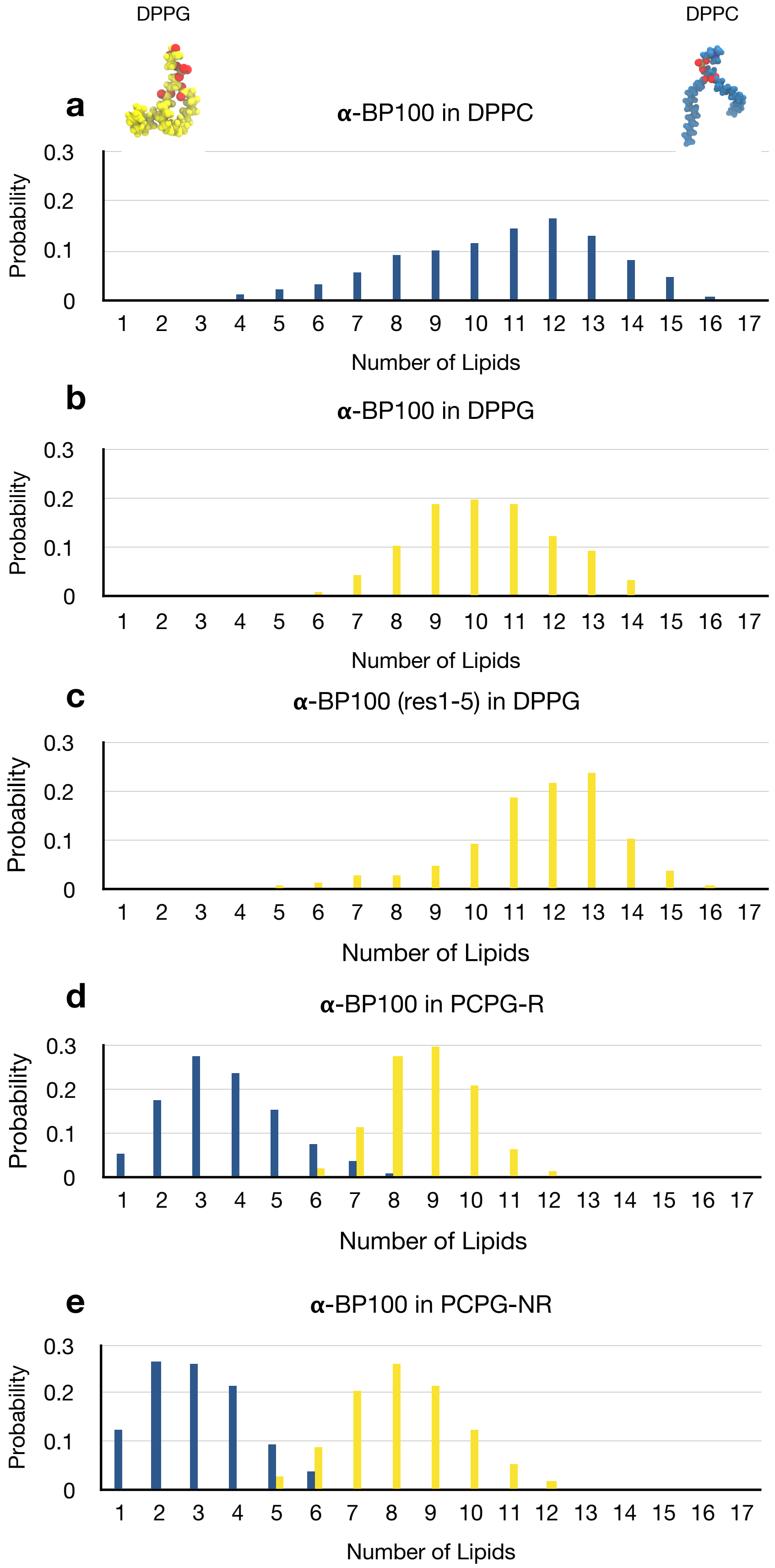

Figure S6) Distribution of lipid in contact with the peptide.

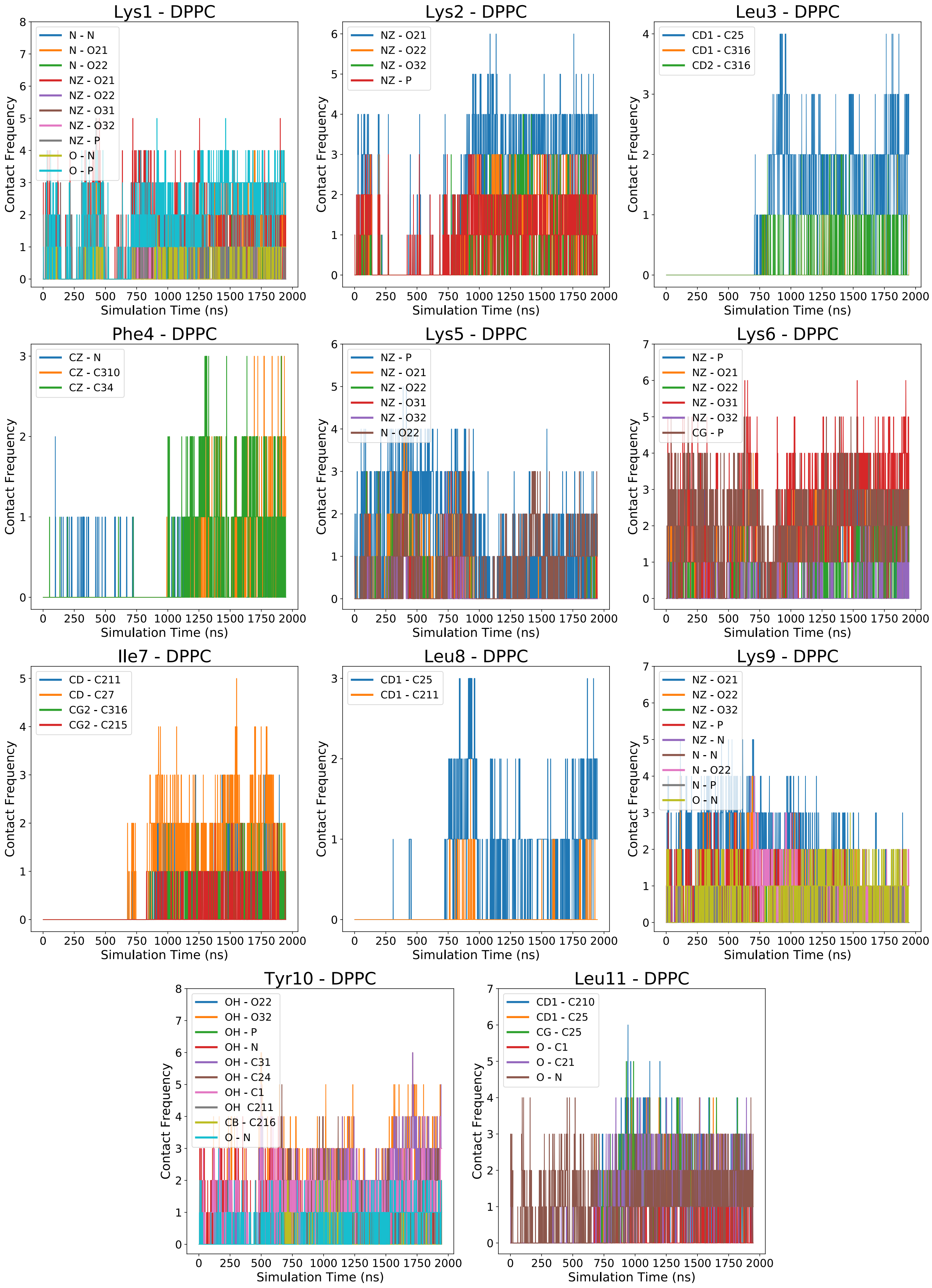

Figure S7) Pair occurrence frequency between BP100 and DPPC for α-BP100 in DPPC simulation. Legends represent a pair between one peptide atom and lipid atom. Peptide flip was observed after *circa* 750 ns of simulation.

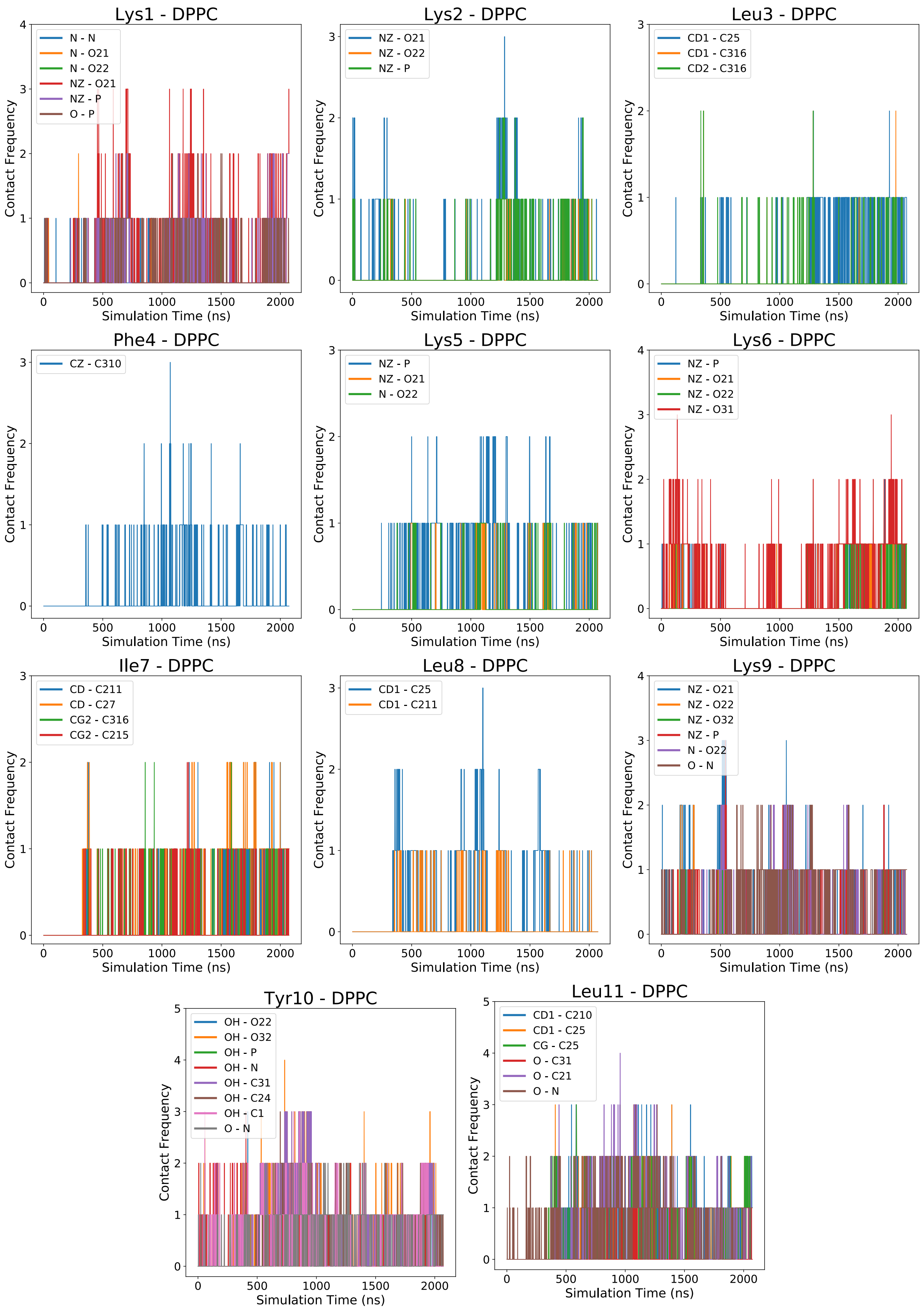

Figure S8) Pair occurrence frequency between BP100 and DPPC for **α-BP100 in PCPG-R** simulation. Legends represent a pair between one peptide atom and lipid atom. Peptide flip was observed approximately after 350 ns of simulation.

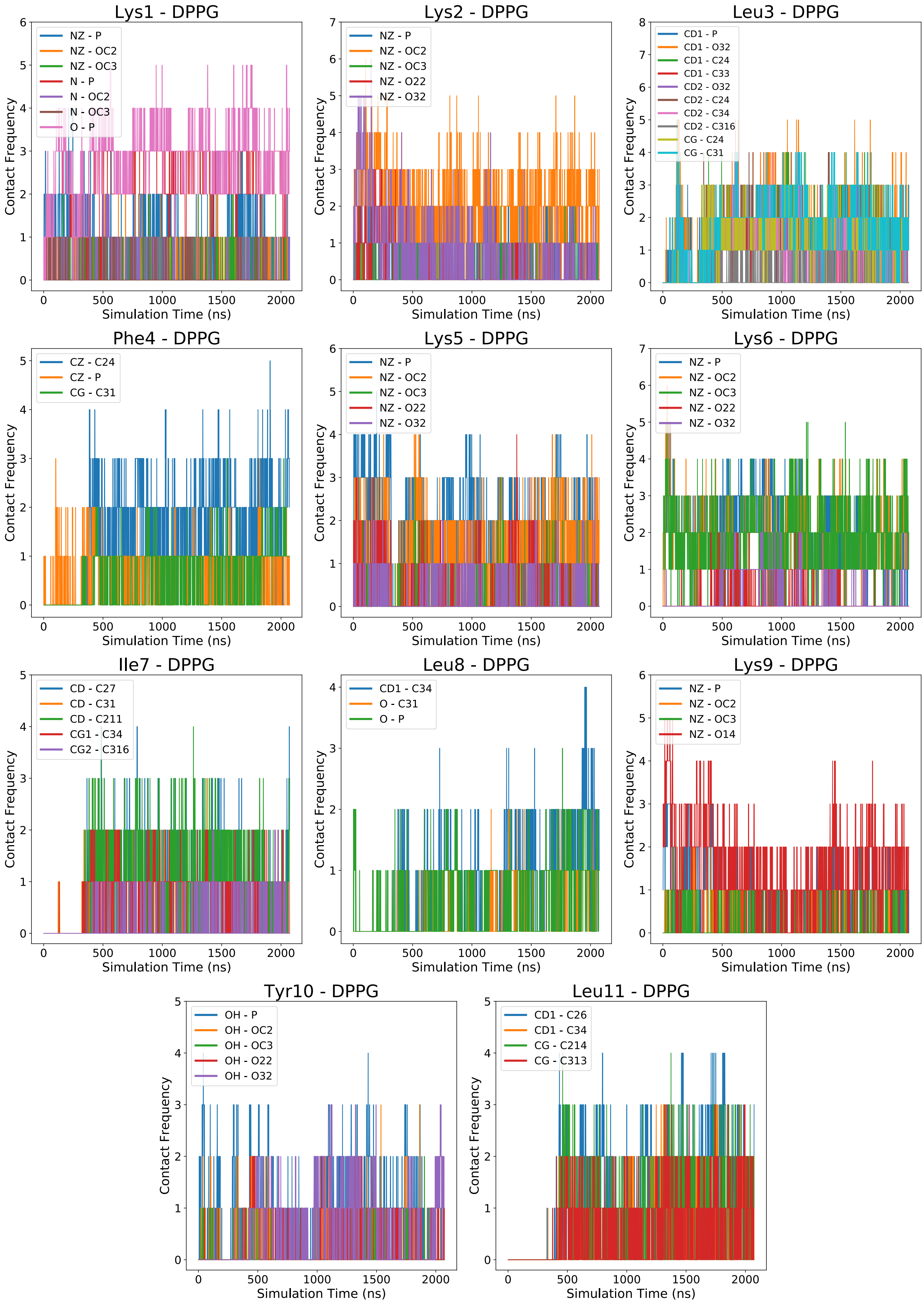

Figure S9) Pair occurrence frequency between <u>BP100 and DPPG</u> for **α-BP100 in PCPG-R** simulation. Legends represent a pair between one peptide atom and lipid atom. Peptide flip was observed approximately after 350 ns of simulation.

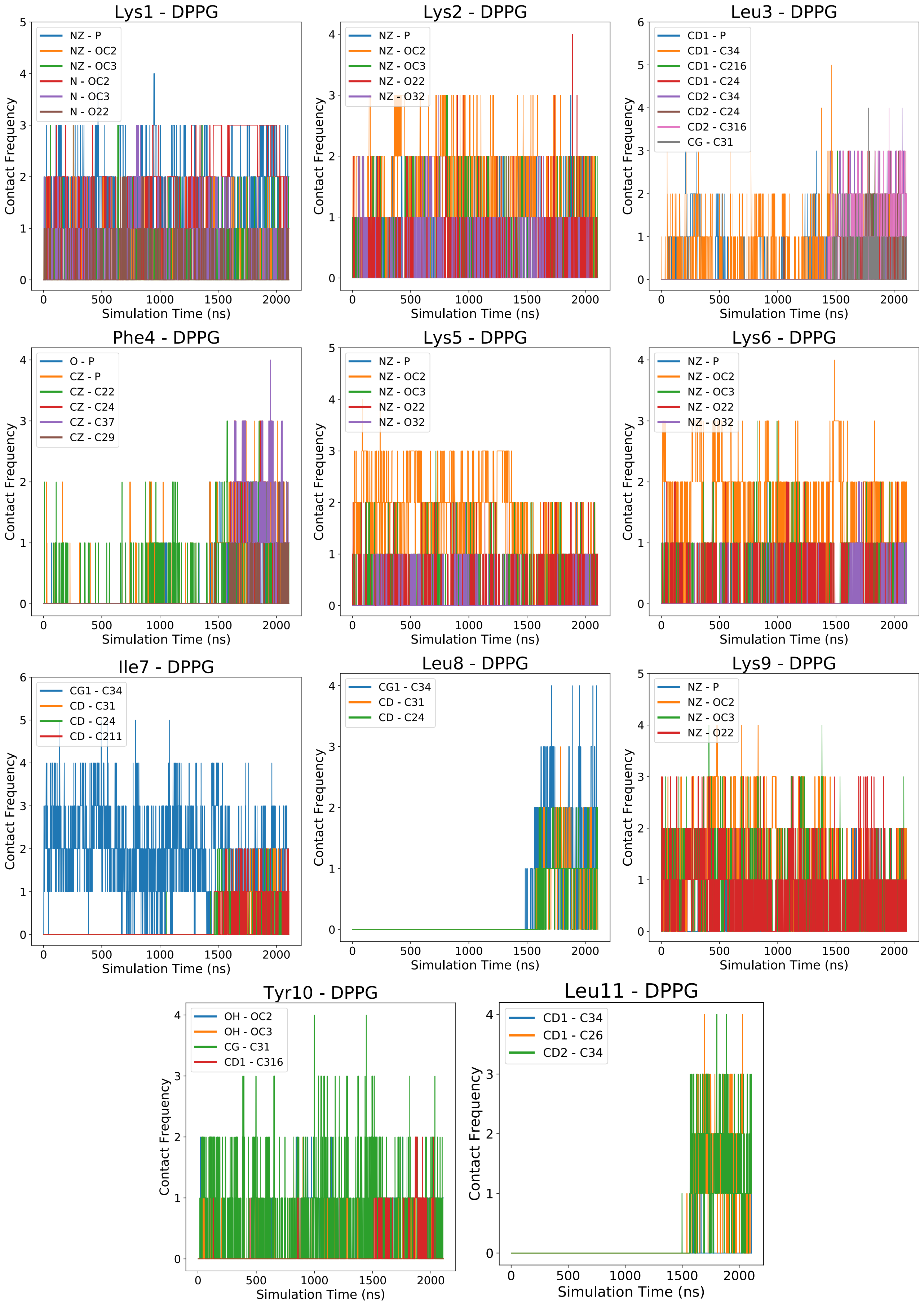

Figure S10) Pair occurrence frequency between <u>BP100 and DPPG</u> for **α-BP100 in DPPG** simulation. Legends represent a pair between one peptide atom and lipid atom. Peptide flip was observed approximately after 1500 ns of simulation.

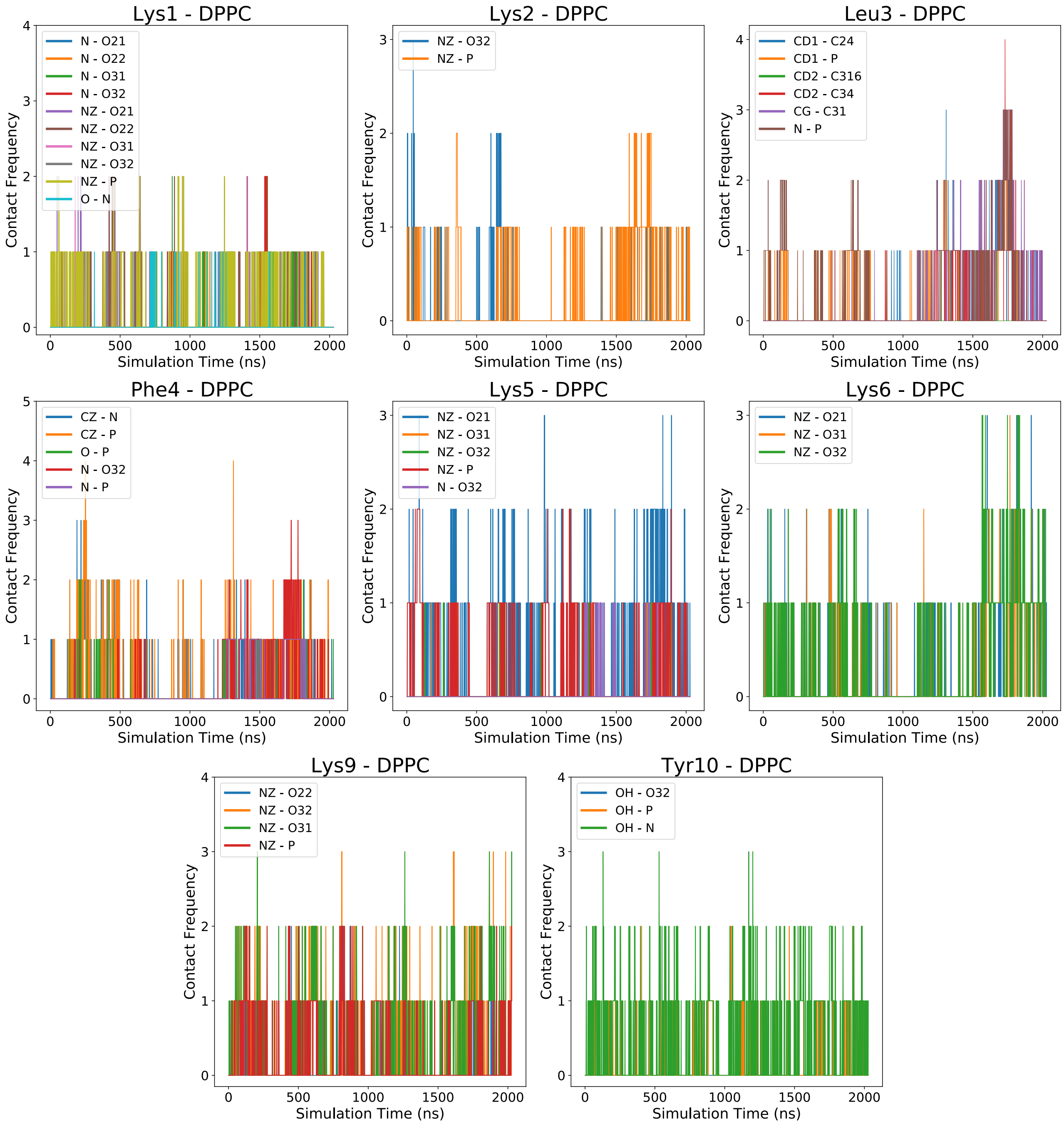

Figure S11) Pair occurrence frequency between BP100 and DPPC for **α-BP100 in PCPG-NR** simulation. Legends represent a pair between one peptide atom and lipid atom. No significant pairs were found for 7Ile, 8Leu and 11Leu residues with BP100. No peptide flip was observed.

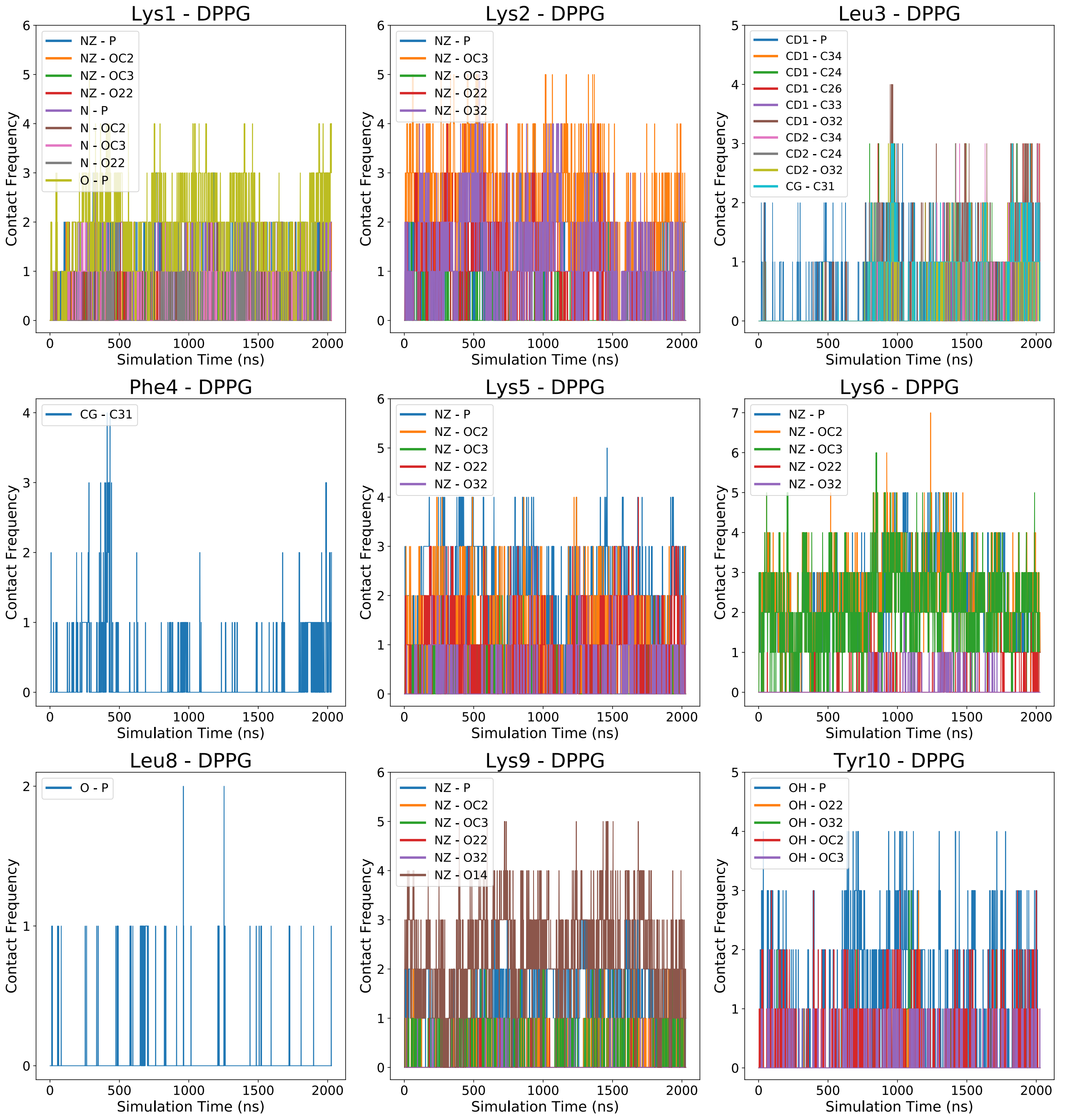

Figure S12) Pair occurrence frequency between BP100 and DPPG for **α-BP100 in PCPG-NR** simulation. Legends represent a pair between one peptide atom and lipid atom. No significant pairs were found for 7Ile, 8Leu and 11Leu residues with BP100. No peptide flip was observed.

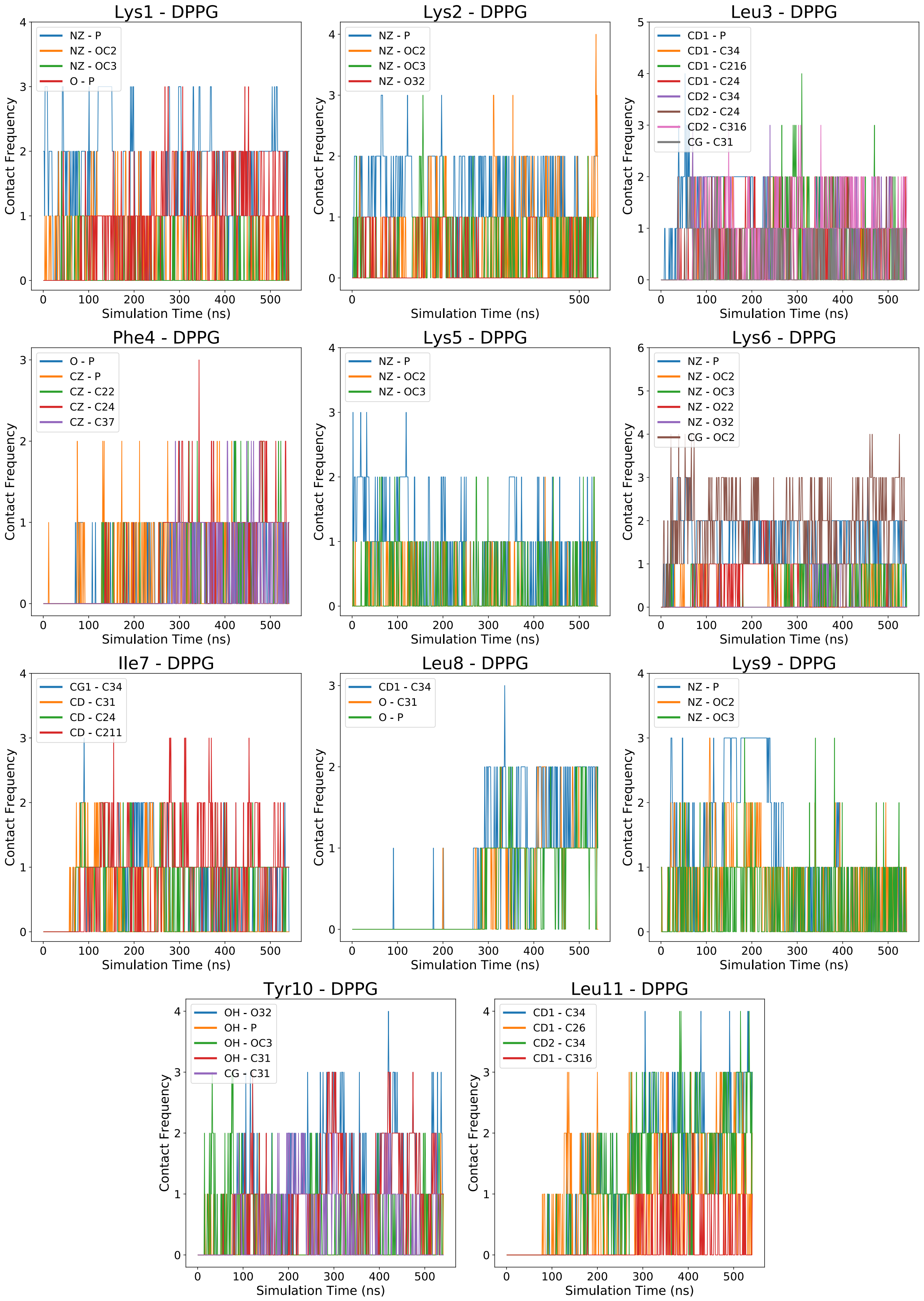

Figure S13) Pair occurrence frequency between BP100 and DPPG for **α-BP100 (res1-5) in DPPG** simulation. Legends represent a pair between one peptide atom and lipid atom. Peptide flip was observed approximately after 300 ns of simulation.

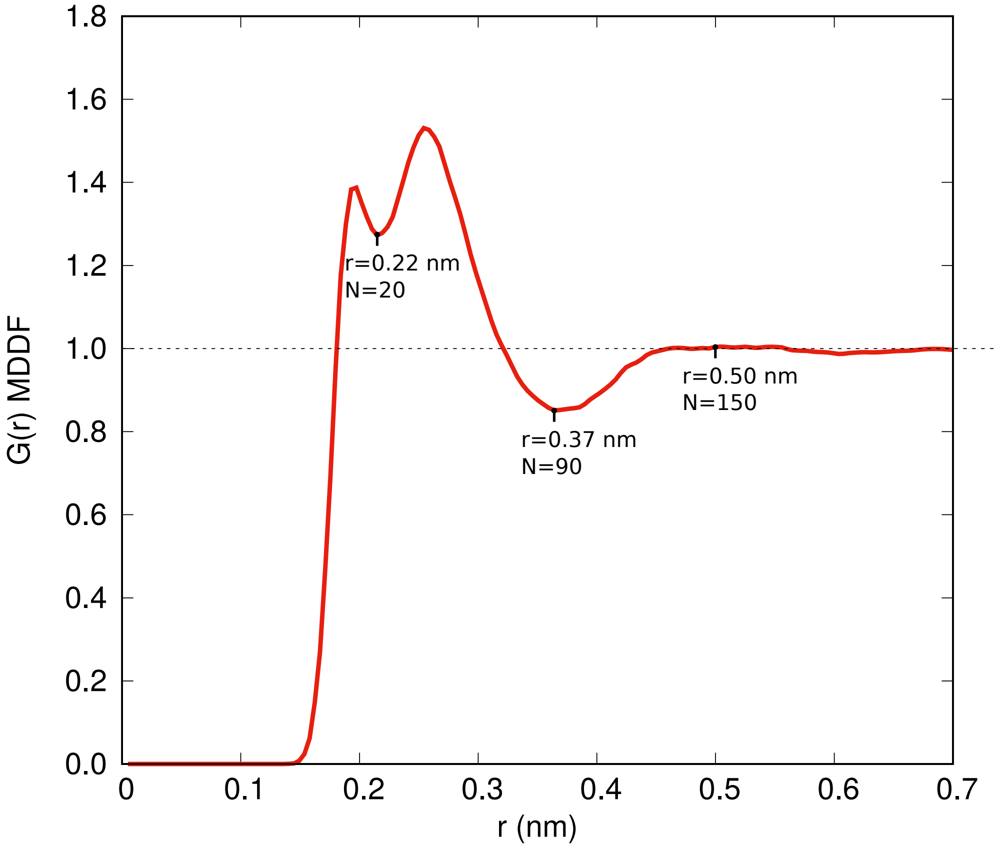

Figure S14) Minimum-distance distribution function (MDDF) between the peptide and the water molecules in the aqueous solution. For our comparative purposes, we first calculated the MDDF of BP100 in aqueous solution. In this distribution we obtained the first solvation shell up to 0.37 nm with 90 water molecules and beyond 0.5 nm no solvation shell could be identified anymore. The amount of water molecules obtained up to 0.5 nm was around 150 and this distance was used to perform further analyses for the comparative hydration of the peptide interacting with the membranes during the flip.

REFERENCES

1. Petrache, H. I. *et al.* Area per Lipid and Acyl Length Distributions in Fluid Phosphatidylcholines Determined by 2H NMR Spectroscopy. *Biophys. J.* **79,** 3172–3192 (2000).
2. Nagle, J. F. *et al.* X-ray structure determination of fully hydrated L alpha phase dipalmitoylphosphatidylcholine bilayers. *Biophys. J.* **70,** 1419–31 (1996).
3. Lis, L. J., McAlister, M., Fuller, N., Rand, R. P. & Parsegian, V. A. Interactions between neutral phospholipid bilayer membranes. *Biophys. J.* **37,** 657–65 (1982).
4. Nagle, J. F. Area/lipid of bilayers from NMR. *Biophys. J.* **64,** 1476–1481 (1993).
5. Kučerka, N., Nieh, M.-P. & Katsaras, J. Fluid phase lipid areas and bilayer thicknesses of commonly used phosphatidylcholines as a function of temperature. *Biochim. Biophys. Acta - Biomembr.* **1808,** 2761–2771 (2011).
6. Jämbeck, J. P. M. & Lyubartsev, A. P. Derivation and Systematic Validation of a Refined All-Atom Force Field for Phosphatidylcholine Lipids. *J. Phys. Chem. B* **116,** 3164–3179 (2012).
7. Tu, K., Tobias, D. J. & Klein, M. L. Constant pressure and temperature molecular dynamics simulation of a fully hydrated liquid crystal phase dipalmitoylphosphatidylcholine bilayer. *Biophys. J.* **69,** 2558–2562 (1995).
8. Kukol, A. Lipid Models for United-Atom Molecular Dynamics Simulations of Proteins. *J. Chem. Theory Comput.* **5,** 615–626 (2009).
9. Pan, J. *et al.* Molecular structures of fluid phase phosphatidylglycerol bilayers as determined by small angle neutron and X-ray scattering. *Biochim. Biophys. Acta - Biomembr.* **1818,** 2135–2148 (2012).
10. Jämbeck, J. P. M. & Lyubartsev, A. P. Another Piece of the Membrane Puzzle: Extending Slipids Further. *J. Chem. Theory Comput.* **9,** 774–784 (2013).
11. Kučerka, N. *et al.* Lipid Bilayer Structure Determined by the Simultaneous Analysis of Neutron and X-Ray Scattering Data. *Biophys. J.* **95,** 2356–2367 (2008).
